# Effects of Packaging-Relevant Contaminants on the Property Evolution and Reprocessability of PET During Repeated Mechanical Recycling

**DOI:** 10.3390/ma19214704

**Published:** 2026-11-05

**Authors:** Roderik Plavec, Slávka Hlaváčiková, Jozef Feranc, Henrieta Suchánková, Zuzana Kramárová, Michal Ďurfina, Leona Omaníková, Mohammadhassan Rahnama Hezaveh, Mária Straková, Nafiseh Babaei, Ján Kruželák, Tibor Dubaj, Pavol Alexy

**Affiliations:** 1 Institute of Natural and Synthetic Polymers, Faculty of Chemical and Food Technology, Slovak University of Technology, Radlinského 9, 812 37 Bratislava, Slovakia; roderik.plavec@stuba.sk (R.P.); slavka.hlavacikova@stuba.sk (S.H.); jozef.feranc@stuba.sk (J.F.); henrieta.suchankova@stuba.sk (H.S.); zuzana.kramarova@stuba.sk (Z.K.); michal.durfina@stuba.sk (M.Ď.); leona.omanikova@stuba.sk (L.O.); mohammadhassan.hezaveh@stuba.sk (M.R.H.); maria.strakova@stuba.sk (M.S.); nafiseh.babaei@stuba.sk (N.B.); jan.kruzelak@stuba.sk (J.K.); 2 Institute of Physical Chemistry and Chemical Physics, Faculty of Chemical and Food Technology, Slovak University of Technology, Radlinského 9, 812 37 Bratislava, Slovakia; tibor.dubaj@stuba.sk

**Keywords:** polyethylene terephthalate (PET), mechanical recycling, polymer contamination, repeated thermomechanical processing

## Abstract

Residual polymeric contaminants from labels, closures, and adhesives can affect the quality and reprocessability of mechanically recycled polyethylene terephthalate (PET). Direct comparison of individual contaminants, however, is often complicated by differences in PET feedstock, contaminant concentration, and processing conditions among published studies. In this work, eight packaging-relevant contaminants—polypropylene (PP), low-density polyethylene (LDPE), high-density polyethylene (HDPE), polystyrene (PS), glycol-modified PET (PETG), poly(lactic acid) (PLA), poly(vinyl chloride) (PVC), and a commercial hot-melt labeling adhesive (HMA)—were compared at a fixed model concentration of 3 wt.% in bottle-grade PET under identical thermomechanical processing conditions. Contaminant selection was supported by ATR-FTIR screening of commercial bottle labels. Melt flow rate, MFR-derived intrinsic viscosity, differential scanning calorimetry, tensile properties, and CIE L*a*b* color characteristics were evaluated over successive processing stages. PET/PP, PET/LDPE, PET/HDPE, PET/PS, and PET/PETG remained processable through three processing stages, whereas PET/PLA and PET/HMA could only be processed through the second stage, and PET/PVC lost stable strand-forming ability after initial compounding. Tg and Tm remained comparatively stable, while crystallization temperature and PET crystallinity showed contaminant- and processing-dependent changes. Elongation at break was generally more sensitive to contamination than tensile strength, and PVC produced the most pronounced discoloration. Industrial PET fractions collected at successive separation and washing/decontamination stages were evaluated as a complementary case study. Progressive purification was associated with improved MFR-derived IV, while TGA showed only minor differences among the fractions. The results demonstrate that contaminant identity strongly affects PET property evolution and reprocessability during repeated mechanical recycling.

## 1. Introduction

Poly(ethylene terephthalate) (PET) is one of the most widely used thermoplastic polyesters in packaging, particularly in beverage bottles because of its transparency, mechanical strength, chemical resistance, processability, and barrier properties [1,2,3,4,5,6]. Its extensive use in short-life packaging has made PET an important material in circular-economy strategies. Mechanical recycling is an established route for post-consumer PET and allows the polymer to remain in relatively high-value applications and closed material loops [7,8,9,10,11]. Bottle-to-bottle recycling is increasingly used to retain PET in packaging applications [10,11,12,13], while recycled PET is also employed in textiles and other secondary products [14,15,16]. Maintaining molecular integrity, processability, optical quality, and mechanical performance during repeated recycling is therefore important for long-term material circularity [10,11,12,13,14,15].

PET is susceptible to thermal, thermomechanical, thermo-oxidative, and hydrolytic degradation during melt processing [17,18,19,20,21,22,23]. Chain scission can reduce molecular weight and intrinsic viscosity (IV), increase melt flow, and modify crystallization, mechanical behavior, and optical properties [24,25,26,27,28,29,30]. Moisture is particularly important because hydrolysis of ester bonds at PET processing temperatures accelerates molecular weight reduction [19,22], while thermal stability is also influenced by competing degradation and stabilization reactions [20,21]. IV is widely used as an indicator of PET processing quality, although its relationship with molecular weight depends on the analytical method and molecular weight distribution [31]. Molecular weight loss may be partly compensated by solid-state polycondensation or reactive chain extension [32,33,34,35,36,37]. The final properties of recycled PET nevertheless reflect the combined effects of molecular degradation, crystallization, morphology, rheological behavior, and processing history [18,24,25,26,27,28,29,32,33].

Post-consumer PET is not an ideal single-polymer feedstock. Its quality depends on package design, collection and sorting routes, previous use, washing efficiency, and the heterogeneity of the incoming material [17,38,39,40]. Recycling streams may contain closures, labels, sleeves, adhesives, other polymers, paper, inks, metals, organic residues, particulates, additives, degradation products, and non-intentionally added substances (NIAS) [41,42,43,44,45,46]. Industrial PET recycling therefore combines several purification and processing operations, including sorting, size reduction, density separation, washing, drying, melt filtration, extrusion, and, where required, additional decontamination or molecular weight restoration steps [3,10,11,17,38,47,48,49]. Complete removal of foreign components is difficult, and residual contaminants may affect the behavior of PET during subsequent melt processing [17,39,40].

Polymeric contaminants are of particular interest because their effects depend on chemical identity, thermal stability, compatibility with PET, and phase morphology. Polyolefins such as PP, LDPE, and HDPE are commonly used in closures, tamper-evident rings, labels, and other packaging components [6,17,40,50]. Their limited compatibility with PET can alter morphology and mechanical behavior even without pronounced chemical degradation of the PET matrix [40,50,51,52]. Alsewailem and Alrefaie reported reduced toughness and moldability in contaminated post-consumer PET and showed that additional processing can further affect mechanical performance [40]. Itim and Philip observed processing-cycle-dependent changes in the crystallization behavior of PP-contaminated PET [52], whereas Vaucher et al. demonstrated that HDPE contamination affects PET morphology and mechanical response as a function of concentration and processing conditions [50]. Thus, polymers originating from similar packaging components may produce markedly different material responses.

Other contaminants introduce different processing challenges. PLA may enter PET recycling streams from biobased packaging, and even relatively small amounts can affect PET rheology and recycling behavior [53]. The need to identify low PLA concentrations in PET streams has also motivated the development of in-line NIR detection methods [54]. At higher PLA fractions, PET/PLA systems show more complex changes in morphology, crystallization, and thermal stability [55]. PVC represents a particularly critical contaminant because it can degrade at PET processing temperatures and generate acidic degradation products that promote PET chain scission and discoloration; detrimental effects have been reported even at low PVC concentrations [56]. Contaminated post-consumer PET has also shown changes in crystallization behavior during repeated processing and subsequent solid-state polycondensation [57]. More generally, foreign functional additives may alter melt stability and mechanical performance in a strongly chemistry-dependent manner [58]. Contaminant identity is therefore an important variable in addition to contaminant concentration.

Labels and adhesives constitute another heterogeneous source of contamination because PET packaging employs different label constructions and adhesive systems [17,59,60]. PET recycling streams may contain PP, PE, PS, PVC, PETG, and adhesive residues originating from wrap-around labels, shrink sleeves, pressure-sensitive labels, and related components [17,59,61]. Studies of mixed label waste have shown the importance of print-layer removal and density-based separation for recovering PET-rich fractions from materials containing PVC and PS [61]. Adhesive residues may remain attached to PET flakes after washing or undergo thermal transformation during melt processing, thereby affecting processability and recyclate quality [17,59,60]. Water- or alkali-removable adhesive systems are consequently preferred from a design-for-recycling perspective, although residual hot-melt adhesives remain relevant contaminants in practical recycling streams [17,60].

Chemical contaminants and NIAS are especially relevant when recycled PET is intended for food-contact applications. They may originate from previous use, PET degradation, additives, printing inks, closures, recycling equipment, or cross-contamination during collection and processing [41,42,43,44,45,46,62,63,64,65,66,67,68,69,70]. Acetaldehyde, benzene, volatile and semi-volatile organic compounds, phthalates, metals, and other organic contaminants have been identified in recycled PET [62,63,64,65,66,67]. Their occurrence depends on feedstock origin and processing history [41,42,43,62,63,64,65], while removal efficiency depends on contaminant properties and the applied recycling and decontamination conditions [47,71]. Washing, extrusion, deodorization, and dedicated decontamination processes can reduce residual contamination [47,48,71]. For food-contact rPET, the effectiveness of these operations is central to safety assessment [41,42,43,68,72]. Current EFSA guidance therefore evaluates post-consumer mechanical PET recycling using challenge tests with surrogate contaminants and defined reference contamination levels [72]. Migration studies likewise emphasize the importance of feedstock quality, residual contamination, and recycled content levels [67,68,69,73], while compounds such as acetaldehyde may additionally be influenced by PET degradation chemistry and scavenger systems [62,74].

Repeated bottle-to-bottle recycling remains technically feasible when feedstock quality, separation, and processing are adequately controlled [10,11,17,73,75]. Modelling of closed recycling loops nevertheless indicates that accumulation of foreign components can limit recycled content levels and final bottle quality [75], and migration studies similarly emphasize the need to maintain sufficient rPET purity at increasing recycled content [73]. Reactive extrusion and solid-state polycondensation can recover part of the molecular properties lost during processing [35,36,37], but such approaches cannot replace the removal of incompatible or thermally unstable contaminants. Efficient sorting, separation, and decontamination therefore remain essential for closed-loop PET recycling [10,11,17,47,71,72,73,75].

An additional difficulty in evaluating contaminated PET is separating the effect of contamination from the effect of repeated processing itself. Controlled studies of bottle-grade PET have shown progressive decreases in IV and molecular weight, increased MFR, changes in crystallization behavior, reduced ductility, and optical changes during repeated processing even without deliberately added contaminants [18,24,27,28,29]. Contaminated systems should therefore be compared with an equivalently processed PET reference so that processing-induced changes can be distinguished from contaminant-specific responses. This distinction is particularly relevant when linking molecular degradation, material properties, heterogeneous feedstocks, and industrial processing conditions [17,18,38].

Despite extensive work on PET contamination, direct comparison among published studies remains difficult. Previous studies have examined heterogeneous post-consumer feedstocks [39,40], PP contamination [52], HDPE [50], PLA [53,54,55], PVC [56], crystallization of contaminated PET [57], PET containing other functional components [58], and recycling issues associated with labels and adhesives [59,60,61]. These studies differ in PET origin and initial quality, contaminant concentration, drying and processing conditions, equipment, residence time, number of processing cycles, and the properties evaluated. As a result, contaminant-specific responses reported in separate studies cannot be compared directly. Systematic studies evaluating a broader set of packaging-relevant contaminants at the same concentration in the same PET matrix and under identical repeated-processing conditions remain limited [17,18,38,39,40,50,52,53,54,55,56,57,58,59,60,61].

The present study therefore compares eight packaging-relevant contaminants—polypropylene (PP), low-density polyethylene (LDPE), high-density polyethylene (HDPE), polystyrene (PS), glycol-modified PET (PETG), poly(lactic acid) (PLA), poly(vinyl chloride) (PVC), and a commercial hot-melt labeling adhesive—at a fixed model concentration of 3 wt.% in the same bottle-grade PET matrix. The selected concentration was used as a comparative challenge level rather than as an estimate of typical residual contamination in industrial rPET. Where processability permitted, the materials were subjected to repeated thermomechanical processing and characterized by melt flow rate (MFR), MFR-derived IV, differential scanning calorimetry, tensile testing, and CIE L*a*b* color analysis. Contaminant selection was supported by ATR-FTIR screening of commercial beverage-bottle labels, and the controlled model experiments were complemented by an industrial PET case study involving successive separation and washing steps. The novelty of the present study lies in the direct side-by-side comparison of eight packaging-relevant contaminants at the same concentration, in the same bottle-grade PET matrix, and under an identical repeated-processing history. This controlled comparison provides practical information on which contaminants most strongly limit PET reprocessability and can therefore support the prioritization of separation, washing, and decontamination steps in industrial PET recycling.

## 2. Materials and Methods

### 2.1. Materials

Bottle-grade polyethylene terephthalate (PET), commercially available as NEOPET 80, manufactured by UAB NEO GROUP (UAB NEO GROUP, Klaipėda, Lithuania) and supplied by General Plastic, a.s. (Kolárovo, Slovakia), was used as the reference polymer matrix. NEOPET 80 is a food-contact PET resin intended for beverage packaging applications and has a nominal intrinsic viscosity of approximately 0.80 dL g^−1^.

Eight polymeric or polymer-based materials relevant to PET packaging waste streams were selected as model contaminants: polypropylene (PP), low-density polyethylene (LDPE), high-density polyethylene (HDPE), polystyrene (PS), glycol-modified polyethylene terephthalate (PETG), polylactic acid (PLA), polyvinyl chloride (PVC), and a commercial hot-melt labeling adhesive. The materials used in this study are summarized in Table 1.

### 2.2. ATR-FTIR Screening of Packaging Labels and Contaminant Selection

Fourteen commercial beverage-bottle labels from different brands available on the Slovak market were screened to support the selection of packaging-relevant model contaminants. The unprinted side of each label was analyzed by attenuated total reflectance Fourier-transform infrared spectroscopy (ATR-FTIR), and the resulting spectra were compared with reference spectral libraries.

Measurements were performed using a Nicolet iS spectrometer equipped with an iD7 ATR accessory and a ZnSe crystal. Spectra were recorded from 500 to 4000 cm^−1^ at a resolution of 4 cm^−1^ using 32 scans. A background spectrum was collected before each measurement.

PP and PS were selected directly on the basis of the label screening. LDPE and HDPE were included as closure-related polyolefins, PETG as a potential label- or sleeve-related polyester, PLA as a potential biobased cross-contaminant, and PVC because of its relevance as a thermally problematic contaminant in PET recycling. TECHNOMELT EM 357 was included as a representative residual hot-melt labeling adhesive.

### 2.3. Preparation of Model PET/Contaminant Blends

Model blends containing 97 wt.% PET and 3 wt.% contaminant were prepared to provide a fixed contamination level for the direct comparison of contaminant-specific effects under identical processing conditions. The selected contaminant concentration was used as a controlled comparative challenge level rather than as a representation of typical residual contamination in industrial recycled PET.

Each contaminated formulation had a total mass of 400 g and consisted of 388 g of NEOPET 80 and 12 g of the respective contaminant. A reference formulation containing 400 g of NEOPET 80 without added contaminant was processed under identical conditions. The investigated systems were designated PET, PET/PP, PET/LDPE, PET/HDPE, PET/PS, PET/PETG, PET/PLA, PET/PVC, and PET/HMA.

Prior to the initial compounding step, PET and all model contaminants except HMA were dried in a vacuum oven at 120 °C for 5 h under reduced pressure (<0.3 atm) to minimize moisture-induced hydrolytic degradation during melt processing. HMA was not subjected to this initial drying step because of its low softening/melting range and was used directly from the original sealed manufacturer packaging.

Compounding was performed using an LTE 16-52 co-rotating twin-screw extruder with a screw diameter of 16 mm and an L/D ratio of 52. The screw speed was maintained at 75 rpm. The temperature profile from the feeding zone toward the die was 5 × 270 °C, 3 × 280 °C, 3 × 270 °C, and 2 × 260 °C. PET and the respective contaminant were premixed before feeding into the extruder. The melt was extruded as strands, air-cooled, and subsequently pelletized.

### 2.4. Repeated Thermomechanical Processing

Repeated processing was carried out using the same twin-screw extrusion conditions as those used for initial compounding.

Before each subsequent processing cycle, all pelletized PET/contaminant blends, including PET/HMA, were dried in a vacuum oven at 120 °C for 5 h under reduced pressure (<0.3 atm).

The residence time during each extrusion pass was approximately 160 s. After extrusion, representative samples were collected for characterization, and the remaining material was air-cooled and pelletized for the next processing cycle.

Processing history was designated as follows:

P1—initial melt compounding;

P2—first reprocessing cycle;

P3—second reprocessing cycle.

The PET reference and PET/PP, PET/LDPE, PET/HDPE, PET/PS, and PET/PETG were processed through P3. PET/PLA and PET/HMA could be processed through P2, whereas PET/PVC could only be processed during P1. Further processing was not possible when the melt no longer retained sufficient melt strength for stable continuous strand formation.

### 2.5. Melt Flow Rate and MFR-Derived Intrinsic Viscosity

Prior to measurement, all samples were dried to constant mass. The melt flow rate (MFR) was determined using a Dynisco Polymer Test LMI 4001 DE plastometer following procedures based on STN ISO 1133-1 [76] and STN ISO 1133-2 [77]. Measurements were performed at 285 °C with a preheating time of 300 s and an applied load of 2.16 kg. The capillary die had a diameter of 2 mm and a length of 8 mm, and MFR was expressed in g/10 min. The same measurement temperature, preheating time, applied load, and die geometry were used for all PET/contaminant systems to ensure direct comparability.

Intrinsic viscosity (IV) was estimated from the measured MFR using the PET conversion provided with the instrument. The same conversion procedure was applied to all samples, and no independent calibration curve was established in the present study. The resulting values are referred to throughout the study as MFR-derived IV and are expressed in dL g^−1^. For the contaminated systems, MFR-derived IV was used as a comparative indicator of melt quality rather than as a direct measurement of the intrinsic viscosity of the PET phase.

Three technical measurements were performed for each material and processing stage (n = 3), and the results are reported as means ± standard deviation.

### 2.6. Differential Scanning Calorimetry

Thermal behavior was investigated using a Mettler-Toledo DSC 1 differential scanning calorimeter. Samples weighing approximately 10–20 mg were sealed in crucibles and analyzed under a nitrogen atmosphere.

The thermal program consisted of equilibration at −20 °C for 2 min, followed by a first heating from −20 to 300 °C at 10 °C min^−1^, an isothermal step at 300 °C for 2 min, cooling from 300 to −20 °C at 10 °C min^−1^, equilibration at −20 °C for 2 min, and a second heating from −20 to 300 °C at 10 °C min^−1^. The thermograms were evaluated using STARe Thermal Analysis Software (version 19.0, Mettler-Toledo GmbH, Schwerzenbach, Switzerland).

The glass transition temperature (T*_g_*), crystallization temperature (T*_c_*), melting temperature (T*_m_*), crystallization enthalpy (Δ*H_c_*), and melting enthalpy (Δ*H_m_*) were determined. Thermal parameters used for comparative evaluation were obtained from the controlled cooling and second-heating scans to minimize the influence of the previous thermal history.

The degree of crystallinity of the PET phase, *χ_c_*, was calculated from the melting enthalpy obtained during the second heating according to

(1) $$\chi_{c}=\frac{\Delta H_{m}}{w_{\mathrm{PET}}\Delta H_{m}^{0}}\times 100$$

where Δ*H_m_* is the measured melting enthalpy of PET, *w*_PET_ is the mass fraction of PET in the formulation (1.00 for the uncontaminated reference and 0.97 for the contaminated blends), and $\Delta H_{m}^{0}=140$ J g^−1^ is the melting enthalpy of fully crystalline PET [26]. The reference enthalpy used in the original measurements was 140 J g^−1^.

One DSC measurement was performed for each formulation and processing stage, and the resulting thermal parameters were evaluated descriptively.

### 2.7. Tensile Testing

Mechanical properties were evaluated using a Zwick/Roell universal testing machine following a procedure based on STN ISO 527 [78] The test specimens consisted of circular strands produced directly during extrusion at the respective processing stages. The strand diameter was approximately 2.0 ± 0.2 mm. For each individual tensile specimen, the diameter was measured at multiple positions along the tested section, and the mean diameter was used to calculate the corresponding circular cross-sectional area.

Tensile testing was performed at a crosshead speed of 50 mm min^−1^. The recorded stress–strain curves were evaluated using testXpert II Testing Software (version 3.71, ZwickRoell GmbH & Co. KG, Ulm, Germany).

Tensile strength at break (σB) and elongation at break (εB) were used as the principal parameters describing the mechanical response. The initial grip separation was 50 mm. Elongation at break (εB) was determined from the strain recorded by the extensometer.

Five strand specimens were tested for each material and processing stage (n = 5), and the results are reported as means ± standard deviation.

### 2.8. Color Characterization

Optical changes induced by contamination and repeated processing were characterized in the CIE L*a*b* color space using a Techkon SpectroDens spectrophotometer. Samples were prepared by extruding a defined amount of material onto a smooth plate and immediately pressing it with a second plate to obtain a reproducible surface suitable for color measurement.

Measurements were performed using D65 illumination, a 10° standard observer, without a polarization filter, and with a 3 mm measurement aperture. A white calibration standard with coordinates L^*^ = 100, a^*^ = 0, and b^*^ = 0 was used.

In addition to the individual L*, a*, and b* coordinates, the total color difference, ΔE*ab, was calculated relative to the white reference standard according to

(2) $$\Delta E_{\mathrm{ab}}^{*}=\sqrt{{\left(L^{*}-L_{\mathrm{ref}}^{*}\right)}^{2}+{\left(a^{*}-a_{\mathrm{ref}}^{*}\right)}^{2}+{\left(b^{*}-b_{\mathrm{ref}}^{*}\right)}^{2}}$$

where $L_{\mathrm{ref}}^{*}=100$, $a_{\mathrm{ref}}^{*}=0$, and $b_{\mathrm{ref}}^{*}=0$. Higher ΔE*_ab_ values therefore indicate a greater overall deviation from the white reference.

Fifteen technical color measurements were performed for each material and processing stage (n = 15).

### 2.9. Industrial PET Recycling Samples

To place the controlled model experiments in an industrial context, PET flakes collected at three successive stages of an industrial bottle recycling process were additionally evaluated.

PET1 consisted of PET flakes containing residual labels and bottle closures before contaminant separation using a ZIG-ZAG separator. PET2 represented PET flakes collected after ZIG-ZAG separation. PET3 represented PET flakes collected after separation followed by an additional alkaline washing/decontamination step using a potassium-hydroxide-based cleaning solution containing detergents. All industrial PET fractions were dried to constant mass before homogenization.

The industrial fractions were homogenized using a Plasticorder Brabender single-screw extruder (Brabender GmbH & Co. KG, Duisburg, Germany) with a screw diameter of 19 mm, an L/D ratio of 25, a screw compression ratio of 1:3, and a circular die with a diameter of 2 mm. The screw speed was 50 min^−1^, and the temperature profile from the feeding zone toward the die was 270, 280, 270, and 260 °C. The extruded strands were subsequently pelletized.

The melt flow rate (MFR) was measured, and the MFR-derived intrinsic viscosity (IV) was calculated using the procedure described in Section 2.5. MFR measurements of the industrial PET fractions were performed under the same temperature, load, preheating time, and die geometry as those used for the model PET/contaminant systems. In the industrial comparison, the MFR-derived IV was used as the principal rheological-quality indicator. The industrial PET fractions were evaluated to contextualize the controlled model-blend experiments and were not treated as direct equivalents or validation samples of the individual laboratory formulations.

Thermogravimetric analysis (TGA) was performed using a Mettler-Toledo TGA/DSC 3+ thermal analysis system (Mettler-Toledo GmbH, Greifensee, Switzerland) over the temperature range of 25–600 °C under a nitrogen atmosphere at a flow rate of 20 mL min^−1^. The samples were heated at a constant rate of 10 K min^−1^. Sample preparation followed the same general crucible-based procedure used for the thermal analysis described in Section 2.6. One TGA measurement was performed for each industrial PET fraction.

## 3. Results and Discussion

### 3.1. Selection and Identification of Packaging-Relevant Contaminants

ATR-FTIR screening of 14 commercial beverage-bottle labels identified polypropylene as the dominant label material within the investigated set. Eleven labels were identified as PP, while one label each was identified as paper, polystyrene, and PET, corresponding to 78.6%, 7.1%, 7.1%, and 7.1% of the analyzed samples, respectively (Table 2). The corresponding ATR-FTIR spectra are provided in Figure S1 in the Supplementary Materials. Because the labels represented a limited set of commercial bottles available on the Slovak market, these results should be regarded as a practical material survey rather than as a representative market analysis. The identified polymer types are nevertheless consistent with materials reported for PET labels and sleeves [17].

PP and PS were therefore included as label-related model contaminants. Paper was not included in the melt-blend experiments because preliminary processing produced an undesirable exothermic response and raised concerns regarding safe operation of the twin-screw extruder.

The model set was extended to include additional contaminants relevant to PET packaging and recycling streams. LDPE and HDPE were selected as representative closure-related polyolefins [6,17,50], PETG as a potential label- or sleeve-related polyester [17], and PLA as a potential biobased cross-contaminant associated with altered PET processing behavior [53,54]. PVC was included because of its well-established detrimental effect during PET melt processing, including degradation and discoloration at PET processing temperatures [56]. A commercial hot-melt labeling adhesive was additionally included to represent residual adhesive associated with bottle labeling systems, since such residues may remain with PET flakes after sorting and washing [17,60].

The final model set comprised eight contaminants with different chemical characteristics, packaging origins, and expected processing behavior. Their comparison at the same 3 wt.% concentration and under identical processing conditions provided the basis for evaluating contaminant-specific effects on PET property evolution and reprocessability.

### 3.2. Rheological Evolution and Processability During Repeated Processing

The rheological response of the PET/contaminant systems during repeated thermomechanical processing is shown in Figure 1 and Figure 2. MFR was measured directly, whereas MFR-derived IV was estimated from the measured MFR using the PET conversion provided with the instrument. The two parameters therefore describe the same underlying melt flow response and were not treated as independent measurements. For the contaminated systems, the measured response reflects the behavior of the blend as a whole and may be influenced by both the PET matrix and the rheological properties of the contaminant phase.

The PET reference showed a pronounced change during repeated processing. MFR increased from 20.7 g/10 min after P1 to 77.2 g/10 min after P3, while MFR-derived IV decreased from 0.837 to 0.638 dL g^−1^. Repeated thermomechanical processing therefore altered PET melt behavior even in the absence of deliberately added contamination. The increase in MFR is consistent with progressive chain scission during repeated thermal and shear exposure, which reduces the average chain length and melt viscosity and consequently facilitates melt flow under the fixed measurement conditions. Similar increases in melt flow and decreases in IV during repeated extrusion of bottle-grade PET have previously been associated with progressive molecular degradation during melt processing [18,24].

The addition of 3 wt.% contaminant modified the rheological response already after P1. All contaminated systems showed higher MFR and lower MFR-derived IV than the equivalently processed PET reference, although the magnitude of the effect varied considerably. PET/PP, PET/LDPE, PET/HDPE, PET/PS, and PET/PETG exhibited P1 MFR values of approximately 46–72 g/10 min and retained sufficient melt strength for processing through P3.

In heterogeneous polymer blends, differences in melt flow cannot be attributed solely to changes in the PET matrix. Component viscosity, compatibility, interfacial interactions, and dispersed-phase morphology may also contribute to the measured response [51]. HDPE, for example, forms an immiscible dispersed phase in PET, and its morphology depends on processing conditions and the rheological properties of both phases [50]. Processing-cycle-dependent changes have likewise been reported for PP-contaminated bottle-grade PET [52]. The rheological trajectories observed here therefore reflect the combined influence of repeated PET processing and contaminant-specific blend behavior.

Repeated processing further differentiated the systems that remained processable through P3. MFR increased, and MFR-derived IV decreased progressively for PET/PP, PET/LDPE, PET/HDPE, PET/PS, and PET/PETG. LDPE- and HDPE-containing systems showed comparatively moderate changes, whereas PET/PP, PET/PS, and PET/PETG exhibited larger increases in melt flow. PET/PETG reached the highest P3 MFR among the systems that remained processable through all three stages. Without direct molecular weight or morphological measurements, the individual contributions of PET matrix changes, contaminant rheology, and phase morphology cannot be separated.

More pronounced changes were observed for PET/PLA and PET/HMA. PET/PLA showed an MFR of approximately 89 g/10 min after P1 and 164 g/10 min after P2, accompanied by a decrease in MFR-derived IV from 0.596 to 0.513 dL g^−1^. Small amounts of PLA have previously been shown to affect PET rheology and recyclability [53]. In the present system, the marked increase in melt flow was accompanied by a loss of sufficient melt strength for further strand processing after P2.

PET/HMA showed an even stronger response, with MFR increasing from approximately 161 g/10 min after P1 to 258 g/10 min after P2 and MFR-derived IV decreasing from 0.515 to 0.459 dL g^−1^. Commercial labeling adhesives may contain polymeric and low-molecular-weight components that influence melt behavior in different ways, and the effect of adhesive residues is strongly dependent on adhesive chemistry and processing conditions [17,60]. As with PET/PLA, stable strand formation could no longer be maintained after P2.

PET/PVC showed the most extreme rheological response. After P1, its MFR reached approximately 2200 g/10 min, compared with 20.7 g/10 min for the PET reference, and the corresponding MFR-derived IV estimate was approximately 0.303 dL g^−1^. Although the individual technical measurements showed substantial scatter, all three values were markedly higher than those obtained for the PET reference and the other investigated systems, confirming the exceptionally high melt flow response of PET/PVC. The material could not form a stable continuous strand and could not be pelletized for further processing. This behavior is consistent with the known thermal instability of PVC at PET processing temperatures, where PVC degradation products can promote further deterioration of PET during melt processing [56]. The 3 wt.% PVC concentration used here should, however, be regarded as a comparative challenge level rather than as a typical residual concentration in industrially sorted rPET.

The achievable number of processing stages therefore provided a direct practical measure of reprocessability. PET/PP, PET/LDPE, PET/HDPE, PET/PS, and PET/PETG remained processable through P3, PET/PLA and PET/HMA through P2, and PET/PVC only through P1. Under otherwise identical drying and extrusion conditions, contaminant identity therefore produced markedly different melt flow trajectories and limits to repeated processing.

### 3.3. Thermal and Crystallization Behavior

The thermal behavior of the PET/contaminant systems after P1, P2, and P3 is shown in Figure 3, Figure 4 and Figure 5. The thermograms were dominated by the characteristic transitions of semicrystalline PET. Additional low-intensity thermal features were observed in some PP-, LDPE-, and HDPE-containing systems and are consistent with the presence of crystalline polyolefin phases at the 3 wt.% contamination level.

Only limited changes were observed in the glass transition and melting regions. Tg remained within a relatively narrow range across the investigated systems and processing stages, while Tm was generally within approximately 250–255 °C. No systematic contaminant-specific shift comparable to the changes observed in melt flow or crystallization behavior was evident. Similar stability of the main PET transition temperatures during repeated processing has been reported previously [24], and comparable behavior has also been observed for HDPE-contaminated PET [50].

Larger differences were observed during controlled cooling. After P1, the PET reference exhibited the lowest crystallization temperature, approximately 183 °C, whereas all contaminated systems crystallized at higher temperatures. PET/PVC showed the largest shift, with Tc reaching approximately 206 °C, while the remaining systems exhibited intermediate values (Figure 6).

The increase in Tc should not be assigned to a single mechanism. In heterogeneous PET systems, crystallization may be influenced by PET chain mobility, heterogeneous nucleation, interfacial effects, dispersed-phase morphology, and the thermal behavior of the contaminant phase [50,52,57]. Itim and Philip, for example, reported an increase in Tc for PP-contaminated PET during repeated extrusion, although the corresponding crystallinity evolution differed from that observed here [52]. Differences between studies are expected because of variations in PET grade, contaminant concentration, processing history, and number of extrusion cycles.

Repeated processing also changed the crystallization behavior of the PET reference. Tc increased from P1 to P3 and approached the values of several contaminated systems at later stages. A similar increase in crystallization tendency during repeated processing of bottle-grade PET has been reported previously and associated with increased mobility of shorter PET chains formed during thermomechanical processing [24]. The convergence of Tc values, however, was not accompanied by equivalent convergence in the other measured properties.

This is also evident from the degree of crystallinity, χc (Figure 7). Values were calculated from the second-heating melting enthalpy and corrected for the PET mass fraction in each formulation. The PET reference showed an overall increase in χc toward P3. PET/PP, PET/LDPE, and PET/PETG followed broadly similar tendencies, whereas PET/HDPE remained comparatively stable and PET/PS did not show a corresponding increase in final crystallinity despite its higher Tc.

The different evolution of Tc and χc indicates that these parameters describe different aspects of crystallization. Tc reflects the temperature at which crystallization proceeds most intensively under the applied cooling conditions, whereas χc reflects the crystalline fraction obtained after the defined thermal history. Previous studies have likewise shown that contamination and reprocessing can modify PET crystallization behavior without producing proportional changes in overall crystallinity [50,57].

PET/PLA exhibited comparatively high Tc and χc after the initial processing stage. PET/PLA blends are known to show concentration- and morphology-dependent changes in crystallization and thermal behavior [55]. In the present system, however, the DSC response cannot be separated from the pronounced changes in melt behavior and limited reprocessability of PET/PLA.

PET/PVC showed the most pronounced thermal response after P1, with both the highest Tc and the highest calculated PET crystallinity. This coincided with its extreme melt flow response and loss of further processability. Dark particles were also observed visually in the PVC-containing material. Such particles may contribute to heterogeneous nucleation, but this mechanism was not directly verified.

Cold crystallization was observed during the first-heating scans, particularly in materials that remained processable through P3. Because this response is strongly influenced by the previous extrusion and cooling history, the comparative evaluation was based primarily on the controlled-cooling and second-heating scans.

### 3.4. Mechanical Properties and Fracture Behavior

The mechanical response of the PET/contaminant systems was evaluated using tensile strength at break (σB) and elongation at break (εB), shown in Figure 8 and Figure 9. PET/PLA, PET/PVC, and PET/HMA are not included because the extruded strands fractured during specimen mounting and valid tensile measurements could not be obtained. Their absence therefore reflects severe embrittlement of these systems rather than missing experimental data.

After P1, the PET reference exhibited the highest tensile strength, approximately 49 MPa, while the mechanically testable contaminated systems showed values mainly in the range of approximately 40–47 MPa (Figure 8). The effect of contamination on σB was therefore comparatively moderate.

A much stronger effect was observed for elongation at break. The PET reference reached approximately 160% after P1, whereas the contaminated systems that could be tested exhibited values of only about 15–35% (Figure 9). At the 3 wt.% model contamination level used here, ductility was therefore more sensitive to contamination than tensile strength. This behavior suggests that the presence of contaminant domains primarily promoted premature failure and loss of deformability, while the load-bearing capacity reflected by tensile strength was affected to a lesser extent.

The pronounced reduction in εB is consistent with the heterogeneous morphology expected for several of the investigated polymer pairs. In immiscible or poorly compatible blends, dispersed contaminant domains may promote stress concentration, interfacial debonding, void formation, and crack initiation. Vaucher et al. reported poorly bonded HDPE inclusions in PET and showed that the resulting mechanical response depends strongly on morphology and processing conditions [50]. Reduced toughness-related properties in contaminated post-consumer PET have likewise been reported by Alsewailem and Alrefaie [40].

Repeated thermomechanical processing produced contaminant-specific and frequently non-monotonic mechanical responses. The PET reference retained comparatively stable tensile strength over the three processing stages, whereas its elongation at break decreased after P1. A stronger reduction in ductility than in tensile strength during repeated processing of bottle-grade PET has also been reported previously [24].

The contaminated systems followed different σB trajectories (Figure 8). PET/PP and PET/LDPE increased after P2 and decreased again after P3; PET/PS showed a gradual reduction, while PET/HDPE reached a higher tensile strength at P3. PET/PETG increased after P2 and subsequently decreased at P3. These differences indicate that the mechanical response cannot be related to melt flow behavior alone and may also depend on phase morphology, interfacial adhesion, crystallinity, defect distribution, and the mechanical contribution of the contaminant phase.

PET/HDPE illustrates this complexity particularly well. Previous work has shown that the mechanical response of PET/HDPE systems depends strongly on processing route, dispersed-phase size, and interfacial morphology [50]. The increase in σB observed here after repeated processing may therefore be associated with changes in blend or specimen structure, although direct morphological evidence was not obtained in the present study.

Elongation at break showed greater variability than tensile strength. PET/PP, PET/LDPE, and PET/HDPE remained substantially less ductile than the PET reference throughout repeated processing. PET/PS and PET/PETG showed higher εB values at P3, but these measurements were accompanied by considerable scatter. They should therefore be interpreted cautiously and do not indicate recovery of the original mechanical performance of PET.

The mechanical response also did not follow the crystallinity trends directly. Changes in χc among the investigated systems were not accompanied by proportional changes in either σB or εB, indicating that crystallinity alone cannot account for the observed mechanical behavior.

The inability to obtain valid tensile measurements for PET/PLA, PET/PVC, and PET/HMA is consistent with their limited processability observed in Section 3.2. These systems showed the strongest loss of melt strength and were also sufficiently brittle to fracture before tensile testing could be completed. Among them, PET/PVC showed the most severe overall response, combining extreme melt flow behavior, altered crystallization, loss of further processability, and pronounced embrittlement.

### 3.5. Optical Properties and Visual Appearance

The visual appearance of the PET reference and PET/contaminant systems is shown in Figure 10, while lightness (L*) and total color difference relative to the white reference (ΔE*ab) are presented in Figure 11 and Figure 12.

Repeated thermomechanical processing affected the optical response of the PET reference even in the absence of added contamination. L* decreased from P1 to P3, while ΔE*ab increased, indicating gradual darkening during repeated processing. Similar changes in optical appearance have previously been reported for repeatedly processed bottle-grade PET [24].

The contaminated systems showed markedly different optical trajectories. PET/LDPE and PET/HDPE exhibited a progressive decrease in L* during repeated processing, accompanied by increasing ΔE*ab. In contrast, PET/PP, PET/PS, and PET/PETG showed non-monotonic changes, indicating that optical behavior was not governed solely by the number of processing cycles.

The measured color response of the heterogeneous systems should not be interpreted as a direct measure of PET molecular degradation. Lightness and total color difference may also be influenced by contaminant dispersion, phase morphology, opacity, and light scattering. These effects are particularly relevant for multiphase blends containing polymers with different refractive and structural characteristics.

PET/PLA showed only moderate changes in optical properties between P1 and P2, with L* decreasing from approximately 58.2 to 55.3 and ΔE*ab increasing from approximately 43.2 to 59.8 and ΔE*ab decreasing from approximately 56.9 to 40.3. These changes did not follow the pronounced rheological deterioration observed for the same systems, indicating that optical response and melt flow behavior evolved independently.

The most pronounced optical change was observed for PET/PVC. After P1, PET/PVC exhibited the lowest L* value (approximately 27.6) and the highest ΔE*ab (approximately 74.4) among all investigated formulations, in agreement with the strong brown discoloration visible in Figure 10. The response is consistent with the known thermal instability of PVC at PET processing temperatures and with previous reports of discoloration in PVC-contaminated PET [56]. The 3 wt.% PVC content used here represents a comparative model challenge level rather than a typical residual concentration in industrial rPET.

The photographs in Figure 10 provide a qualitative comparison of the visible changes among the investigated systems. They were not used for quantitative color evaluation. Missing P2 and P3 images for PET/PVC and missing P3 images for PET/PLA and PET/HMA correspond to formulations that could no longer be processed to those stages.

### 3.6. Industrial Context: Effect of Separation and Washing on rPET Quality

The industrial PET fractions provided a practical comparison with the controlled model systems. PET1 represented material collected before ZIG-ZAG separation, PET2 after ZIG-ZAG separation, and PET3 after subsequent alkaline washing/decontamination.

A progressive reduction in visually identifiable foreign material was observed from PET1 to PET3 (Figure 13). PET1 contained numerous colored and printed fragments associated with non-PET packaging components. After ZIG-ZAG separation, the amount of visible foreign material was reduced, while PET3 consisted predominantly of transparent PET flakes with only a limited number of visually detectable contaminants. The photographs provide a qualitative indication of progressive purification and were not used for quantitative assessment of contaminant content.

Thermogravimetric analysis showed only small differences among the industrial fractions (Figure 14). PET1 exhibited a slightly earlier mass-loss response, whereas the main degradation regions of PET1, PET2, and PET3 largely overlapped. Differences between PET2 and PET3 were particularly small. Under the present measurement conditions, bulk TGA therefore did not clearly resolve differences among the successive purification stages.

The limited separation of the TGA curves is consistent with the PET-rich composition of all three fractions, for which the overall mass-loss response was dominated by the PET matrix. Residual packaging polymers or other foreign components may also degrade within overlapping temperature ranges, limiting their distinction by bulk thermogravimetric analysis.

A clearer difference was observed in the MFR-derived IV values (Figure 15). PET1 and PET2 showed similar values of approximately 0.71–0.72 dL g^−1^, whereas PET3 reached approximately 0.755 dL g^−1^. The virgin PET reference exhibited an MFR-derived IV of approximately 0.84 dL g^−1^. The higher value obtained for PET3 indicates that the more extensively purified fraction had a rheological response closer to that of the virgin PET reference.

The increase in MFR-derived IV after washing/decontamination should not be interpreted as restoration of previously degraded PET chains. PET3 represents a different, more extensively purified fraction of the industrial stream. The observed change is therefore more appropriately associated with differences in feedstock composition and residual contamination than with molecular repair.

The industrial results complement the controlled model experiments but should not be considered a direct validation of the individual laboratory blends. The model systems isolate contaminant-specific effects under fixed conditions, whereas PET1–PET3 represent heterogeneous post-consumer fractions containing an unknown combination of labels, closures, adhesives, particulates, and other residual components.

From an industrial perspective, these results support the practical importance of stepwise separation and washing before melt reprocessing, as improved feedstock purity can contribute to more consistent recyclate quality. In addition to the material property changes evaluated here, residual organic contaminants may also contribute to odor formation during thermal processing. Their removal can involve efficient washing or extraction, thorough drying, melt degassing under vacuum, and, where necessary, dedicated deodorization steps [48].

## 4. Conclusions

Eight packaging-relevant contaminants were compared at a fixed model concentration of 3 wt.% in the same bottle-grade PET and under identical thermomechanical processing conditions. The results showed a strong dependence of PET property evolution and reprocessability on contaminant identity.

The most pronounced differences were observed in melt behavior and the ability to undergo repeated processing. PET/PP, PET/LDPE, PET/HDPE, PET/PS, and PET/PETG remained processable through P3, whereas PET/PLA and PET/HMA could only be processed through P2, and PET/PVC lost stable strand-forming ability after P1. MFR-derived IV followed the corresponding melt flow response and was used as a comparative indicator of melt quality rather than as a direct measurement of the intrinsic viscosity of the PET phase.

The effects of contamination were also reflected in thermal, mechanical, and optical properties. Tg and Tm remained comparatively stable, while Tc and PET crystallinity showed contaminant- and processing-dependent changes that did not evolve in parallel. Elongation at break was generally more sensitive to contamination than tensile strength, and PVC produced the most pronounced discoloration. These results show that the response of contaminated PET during repeated processing cannot be described by a single material parameter.

The industrial PET fractions provided a complementary process-scale comparison and showed the practical importance of progressive separation and washing/decontamination for improving feedstock quality. At the fixed contamination level investigated here, contaminant identity determined whether repeated processing resulted in moderate property changes or a loss of further processability. The results therefore support contaminant-specific control and effective purification of PET recycling streams, particularly with respect to PVC, residual labeling adhesives, and PLA cross-contamination. Further work at lower, industrially representative contaminant levels, combined with direct molecular weight and morphological characterizations, would help clarify the contributions of PET matrix degradation, contaminant rheology, and phase morphology.

## Figures and Tables

**Figure 1:**
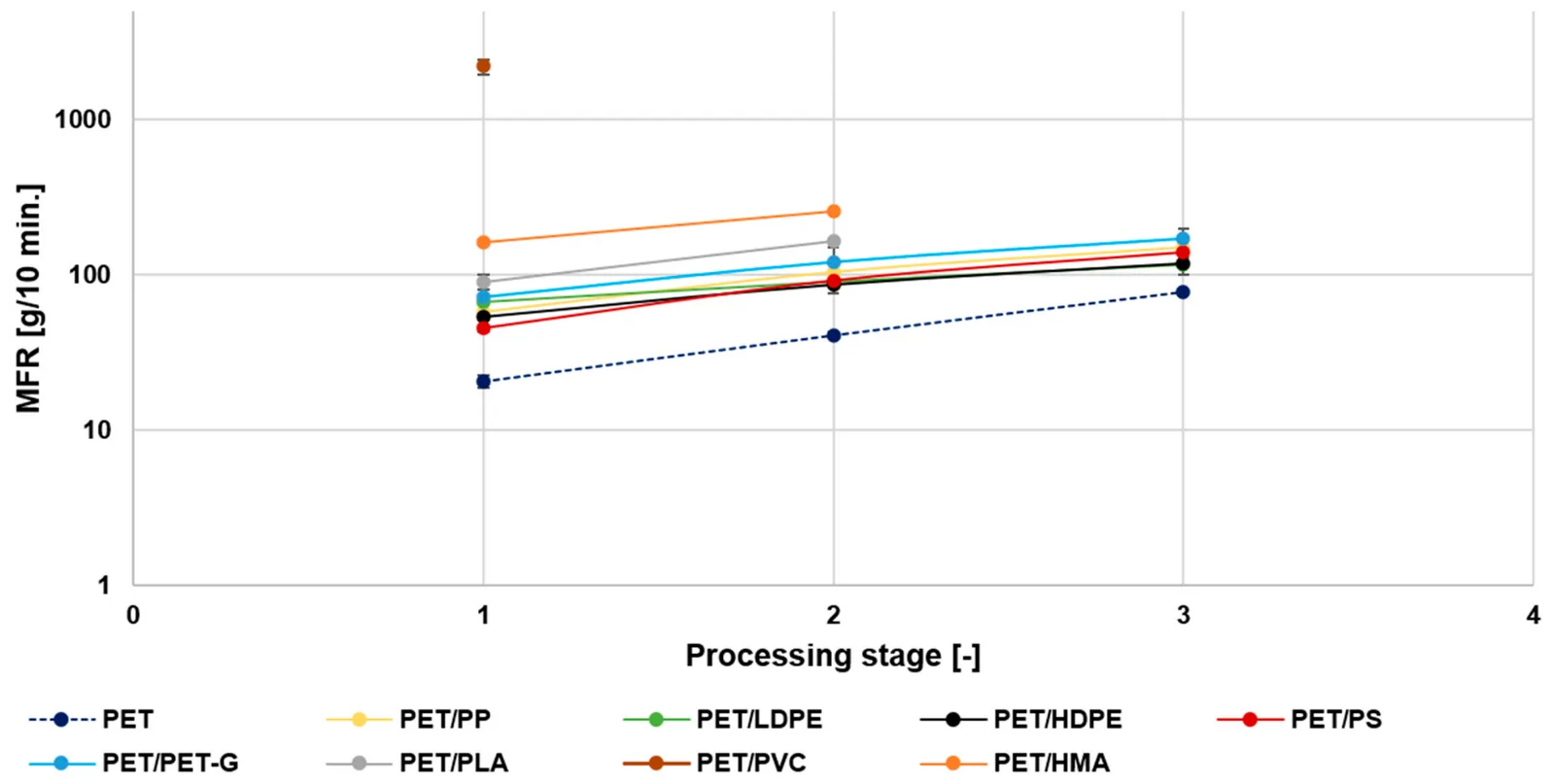
Melt flow rate (MFR) of the PET/contaminant systems during repeated thermomechanical processing. P1 represents the initial melt compounding step, P2 the first reprocessing cycle, and P3 the second reprocessing cycle. The *y*-axis is presented on a logarithmic scale. Values are presented as means ± standard deviation (n = 3 technical measurements).

**Figure 2:**
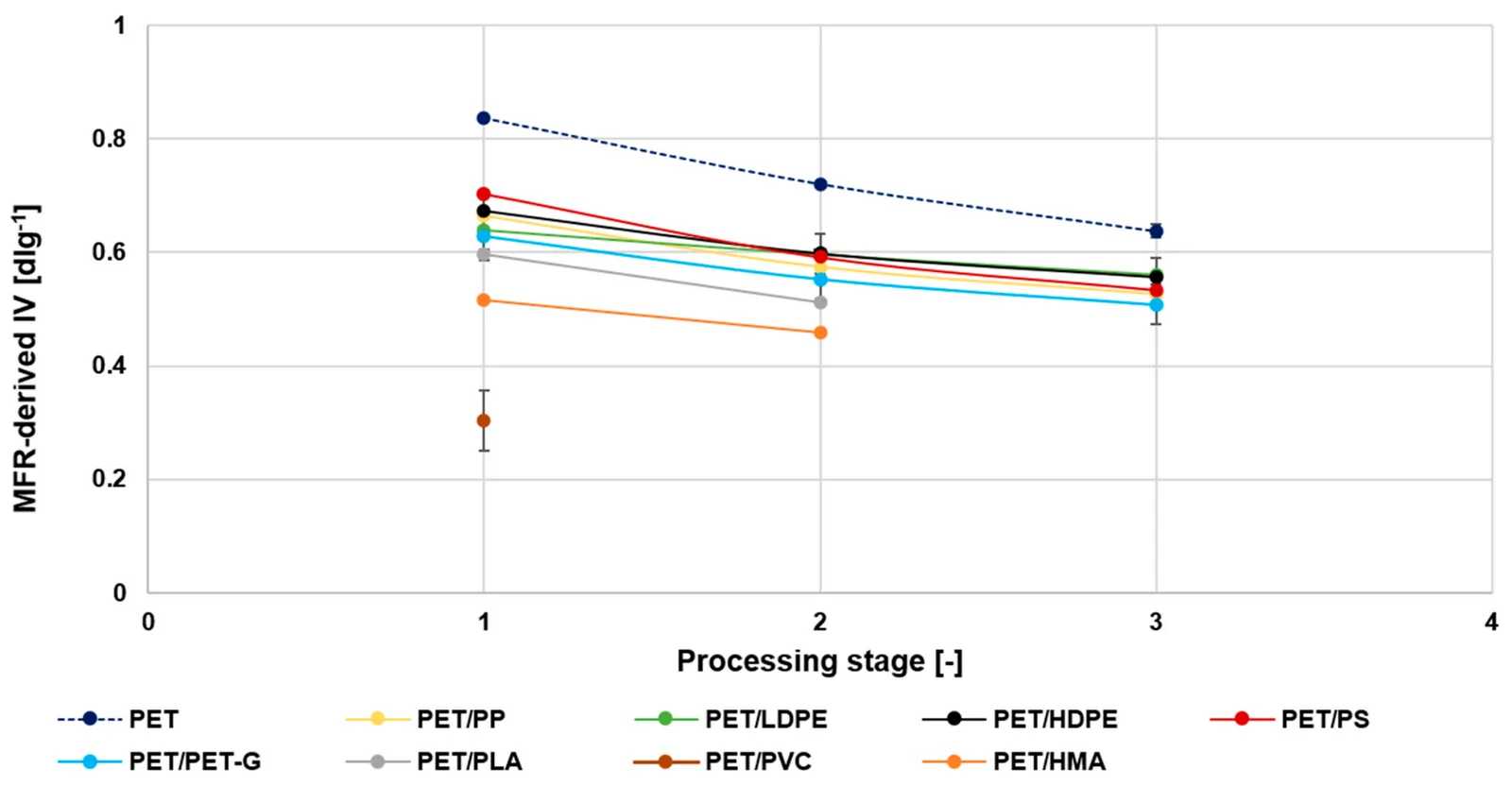
MFR-derived intrinsic viscosity (IV) of the PET/contaminant systems during repeated thermomechanical processing. P1 represents the initial melt compounding step, P2 the first reprocessing cycle, and P3 the second reprocessing cycle. Values are presented as means ± standard deviation (n = 3 technical measurements).

**Figure 3:**
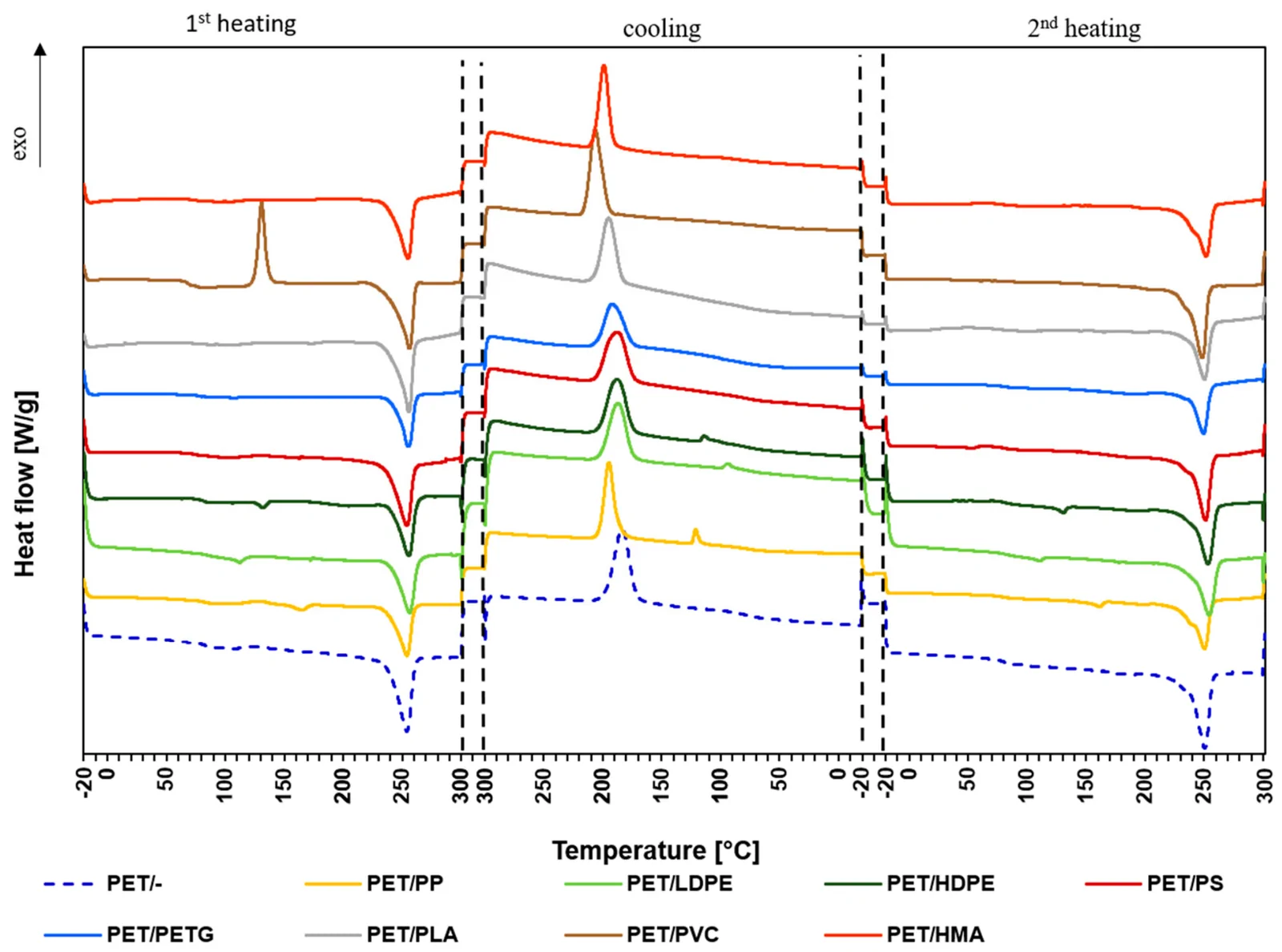
DSC thermograms of the PET reference and PET/contaminant systems after the initial melt compounding stage (P1), showing the first-heating, controlled-cooling, and second-heating segments of the thermal program.

**Figure 4:**
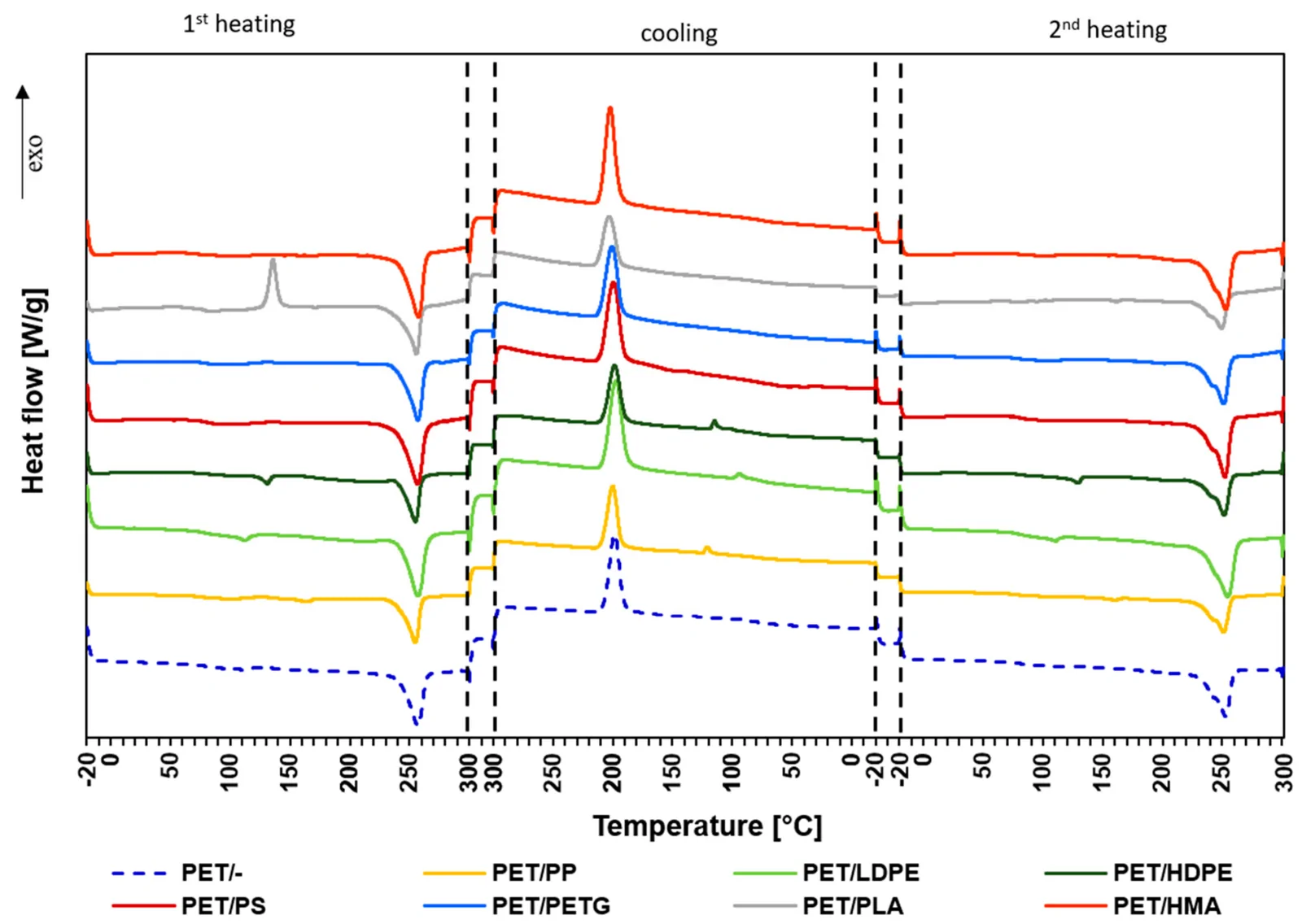
DSC thermograms of the PET reference and PET/contaminant systems after the first reprocessing cycle (P2), showing the first-heating, controlled-cooling, and second-heating segments of the thermal program.

**Figure 5:**
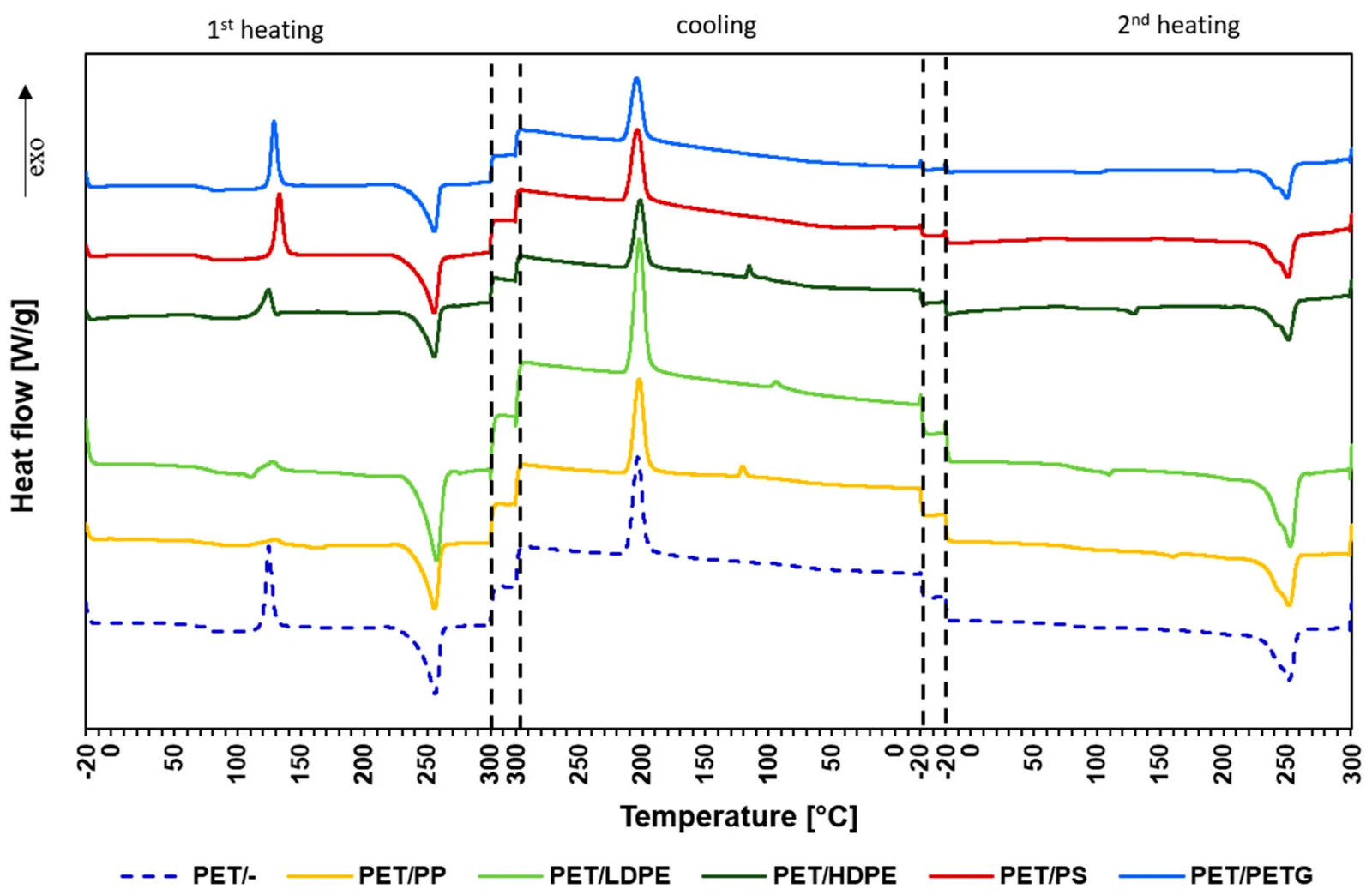
DSC thermograms of the PET reference and PET/contaminant systems after the second reprocessing cycle (P3), showing the first-heating, controlled-cooling, and second-heating segments of the thermal program.

**Figure 6:**
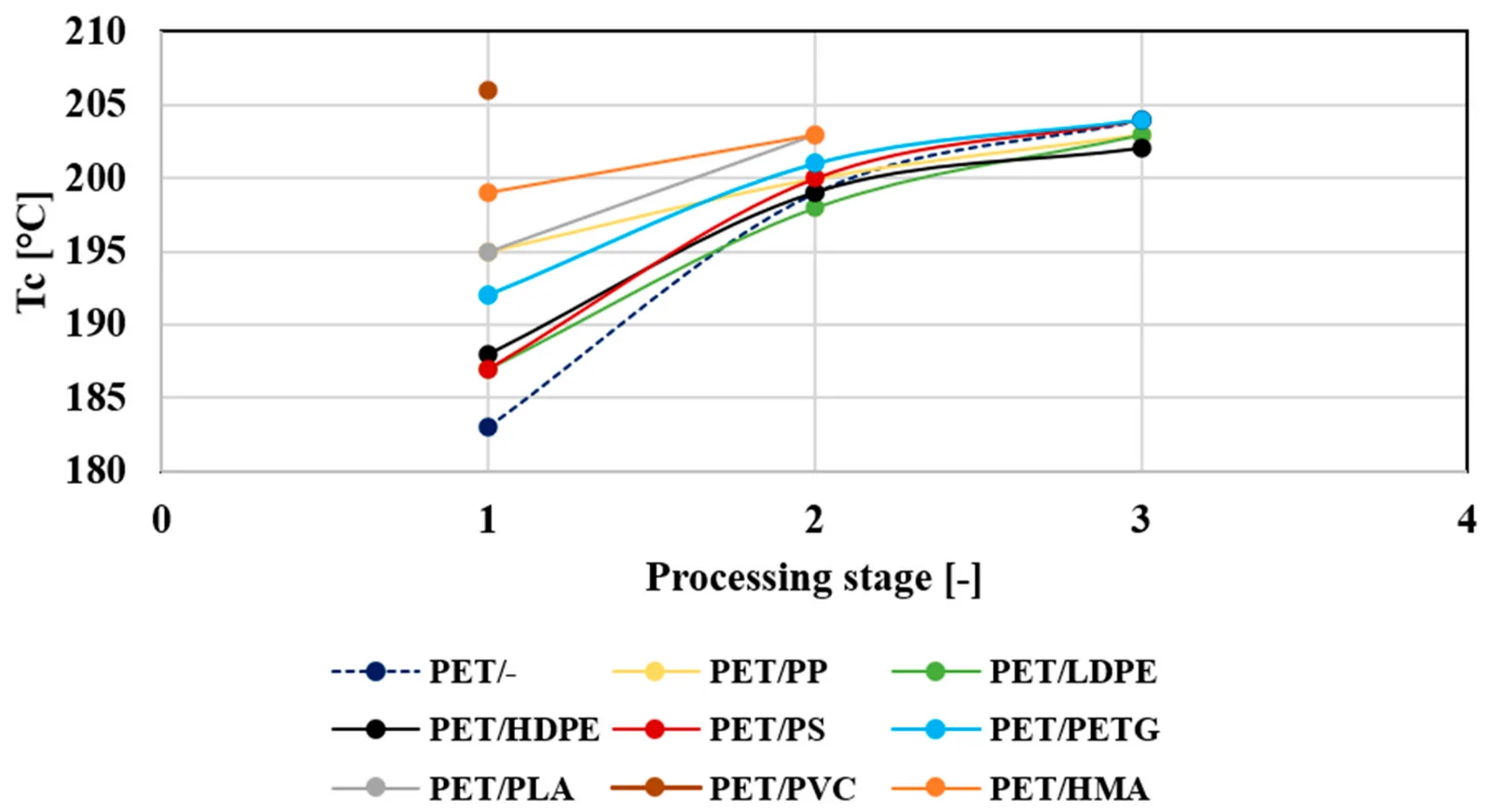
Crystallization temperature (Tc) of the PET reference and PET/contaminant systems as a function of processing stage. Tc values were determined from the controlled-cooling scans.

**Figure 7:**
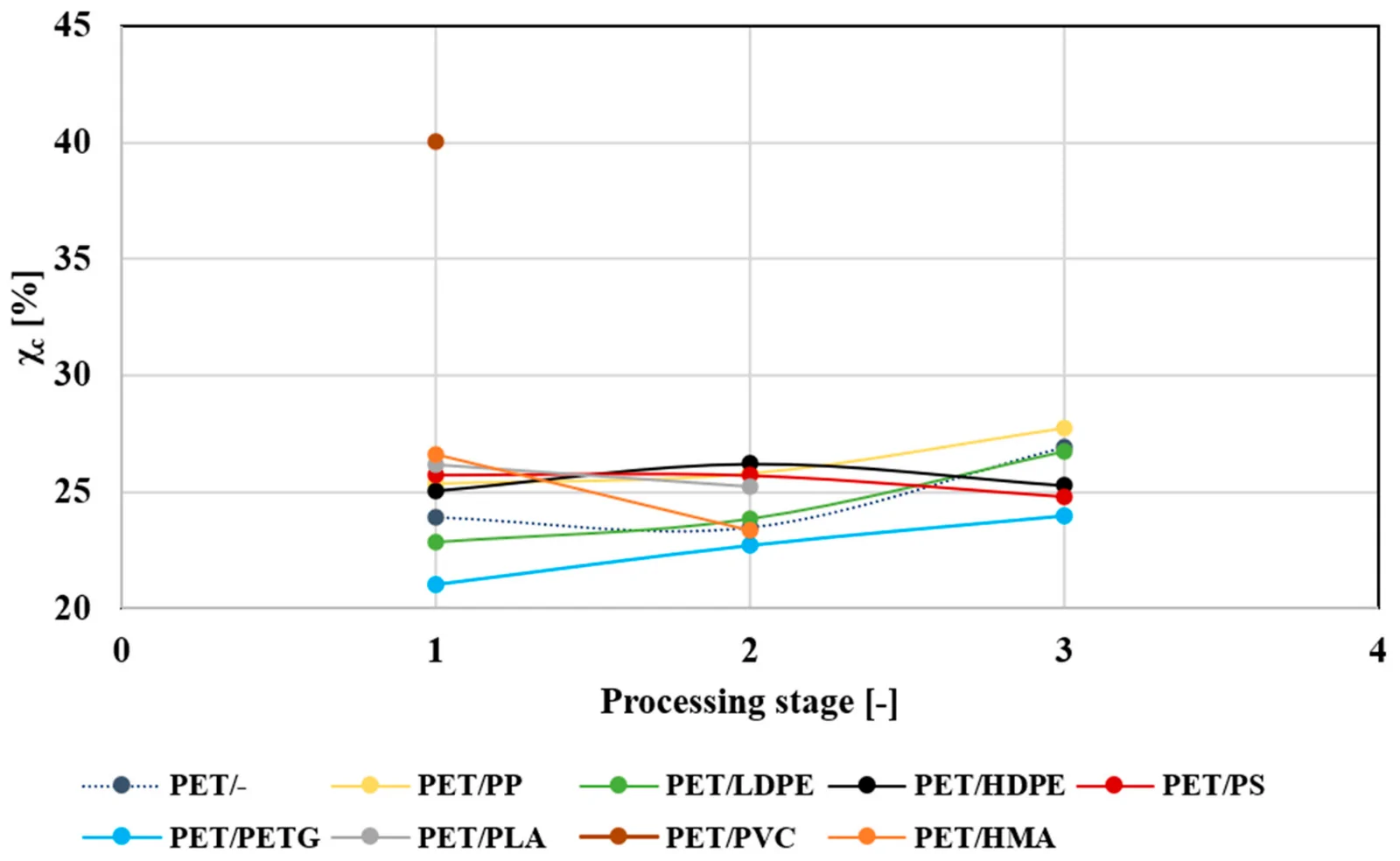
Degree of crystallinity of the PET phase (χc) as a function of processing stage, calculated from the second-heating melting enthalpy and corrected for the PET mass fraction in each formulation.

**Figure 8:**
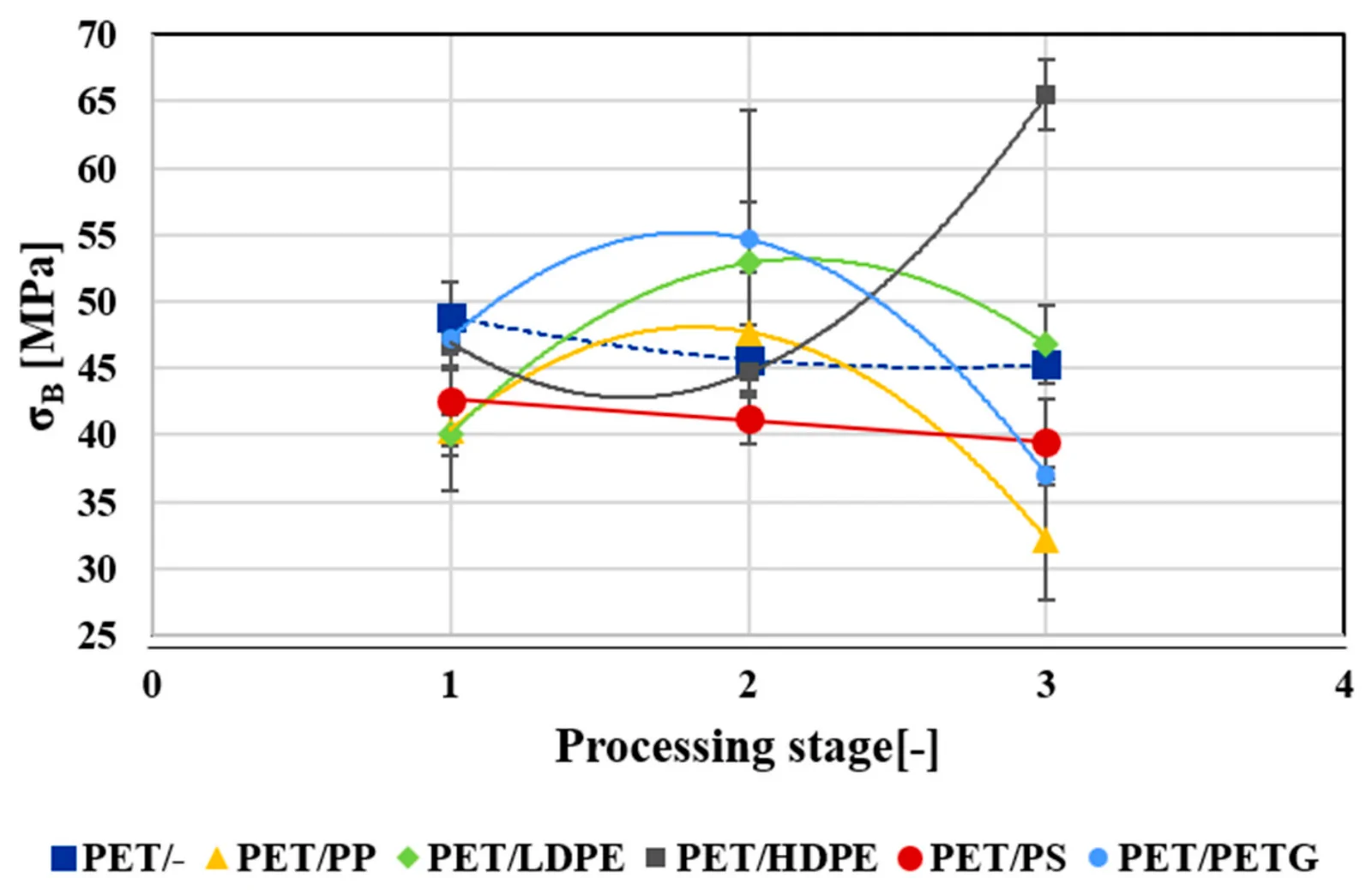
Tensile strength at break (σB) of the PET reference and mechanically testable PET/contaminant systems as a function of processing stage.

**Figure 9:**
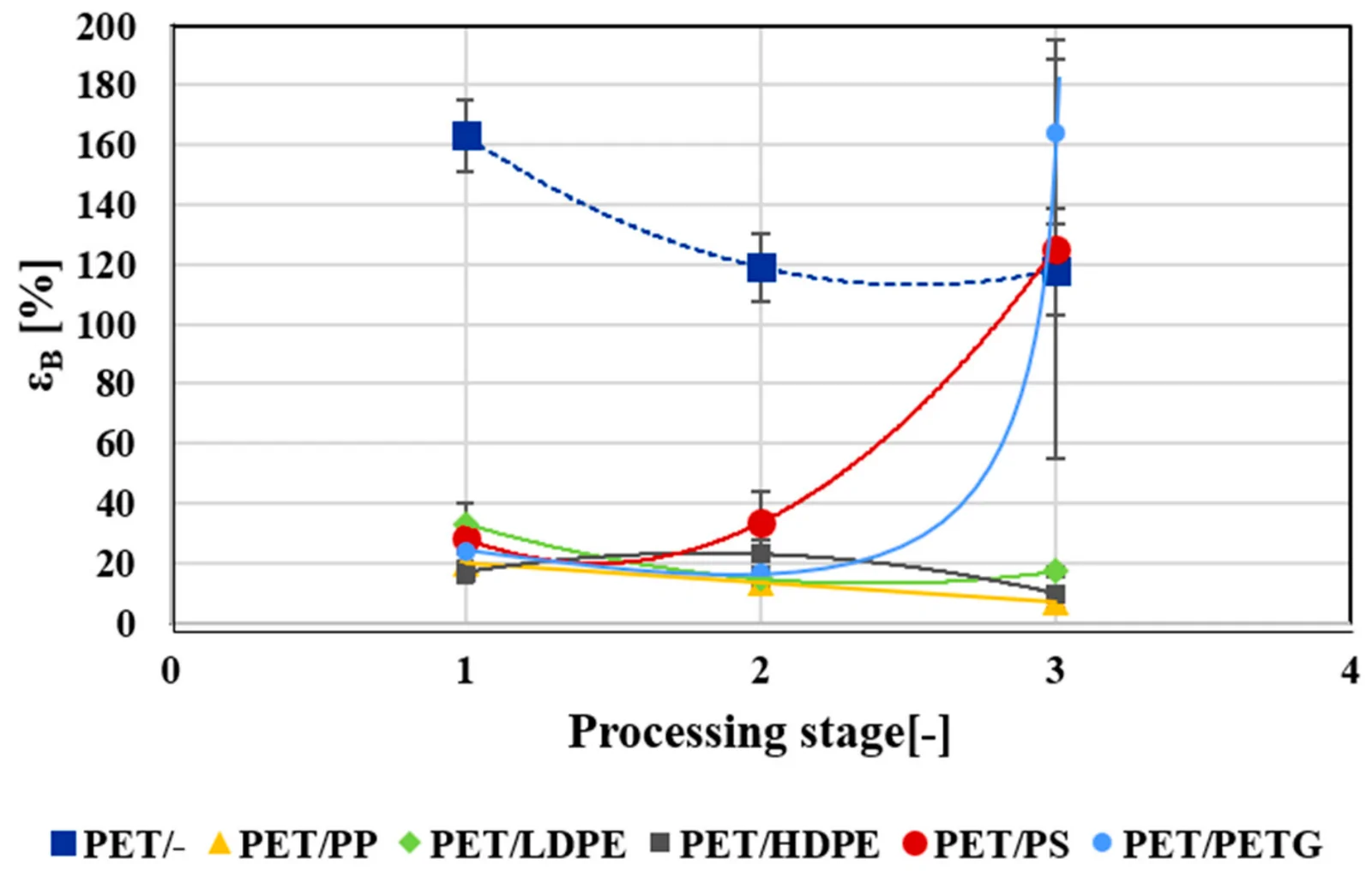
Elongation at break (εB) of the PET reference and mechanically testable PET/contaminant systems as a function of processing stage.

**Figure 10:**
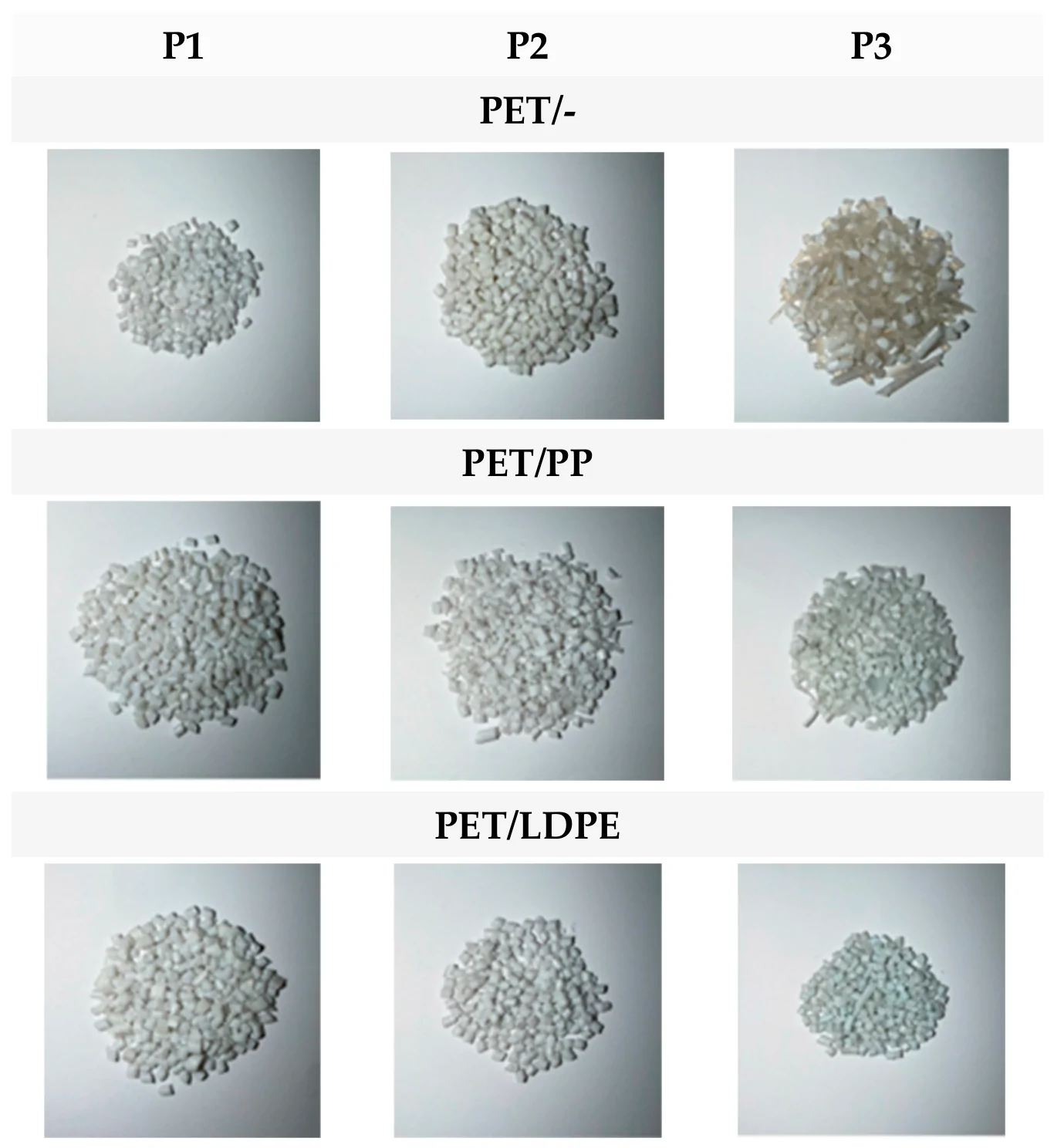

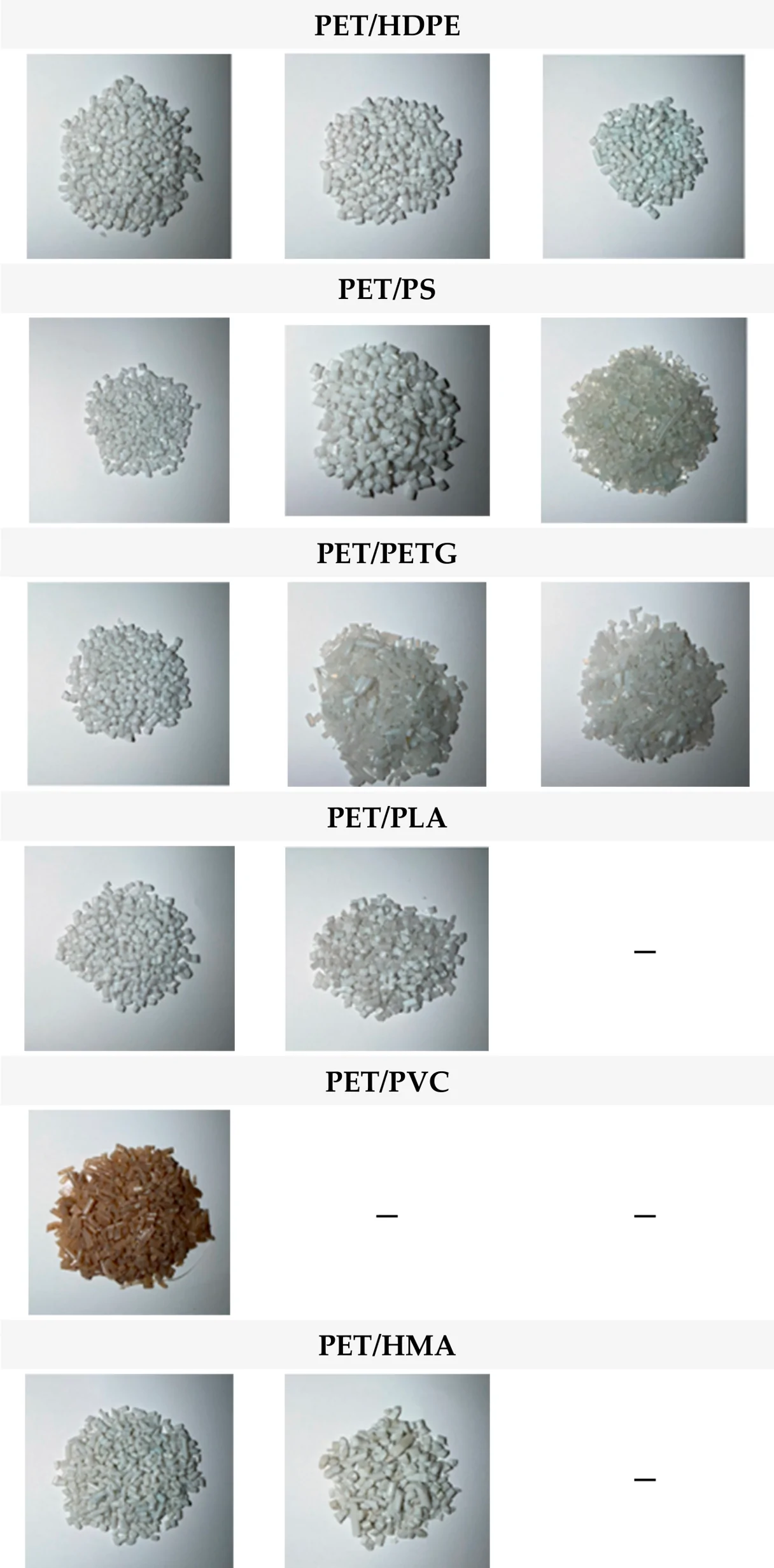
Visual appearance of the PET reference and PET/contaminant systems after successive thermomechanical processing stages. P1 represents the initial melt compounding step, P2 the first reprocessing cycle, and P3 the second reprocessing cycle.

**Figure 11:**
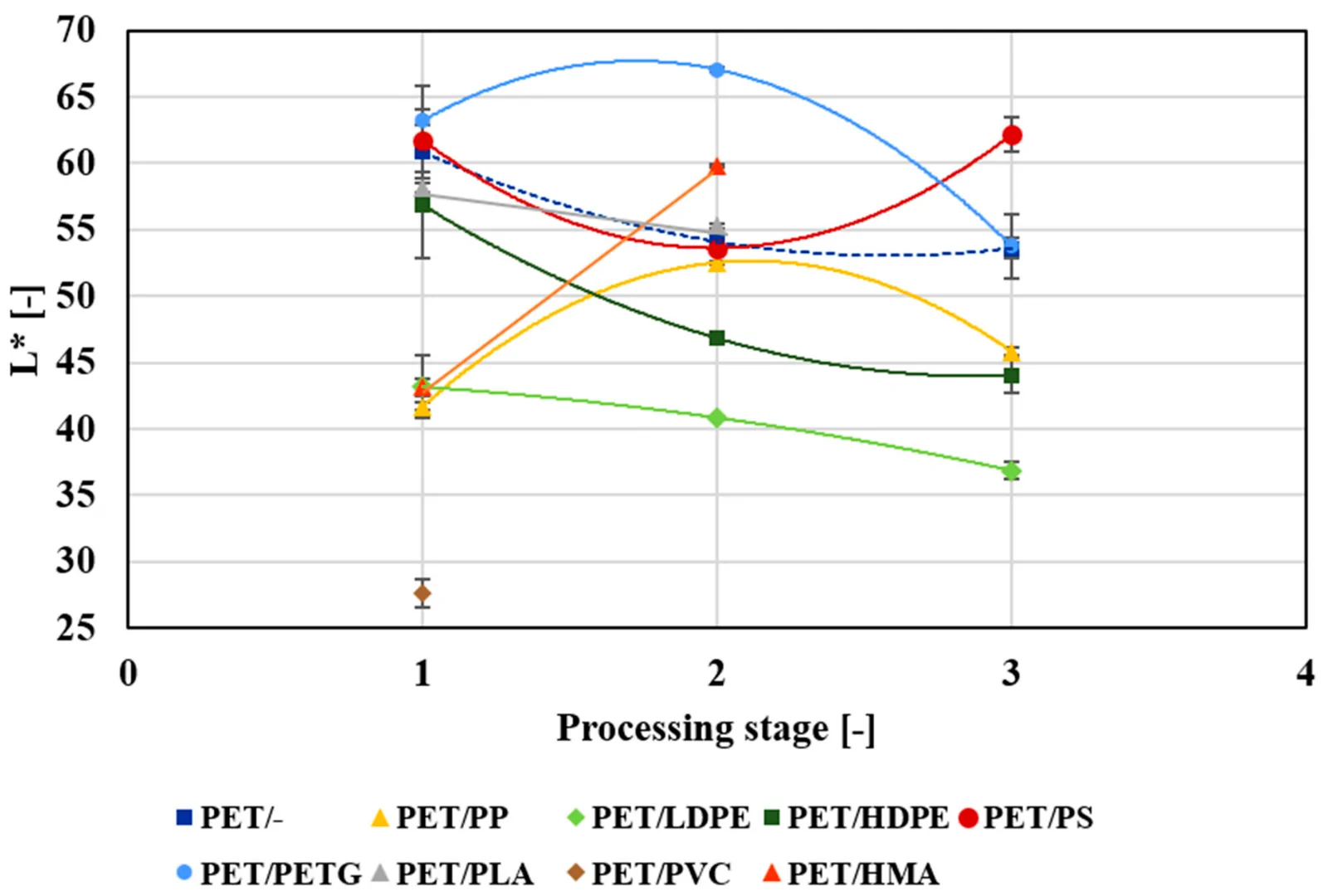
Lightness (L*) of the PET reference and PET/contaminant systems as a function of processing stage. Higher L* values correspond to lighter material. Values are presented as means ± standard deviation. Fifteen technical measurements were performed for each material and processing stage.

**Figure 12:**
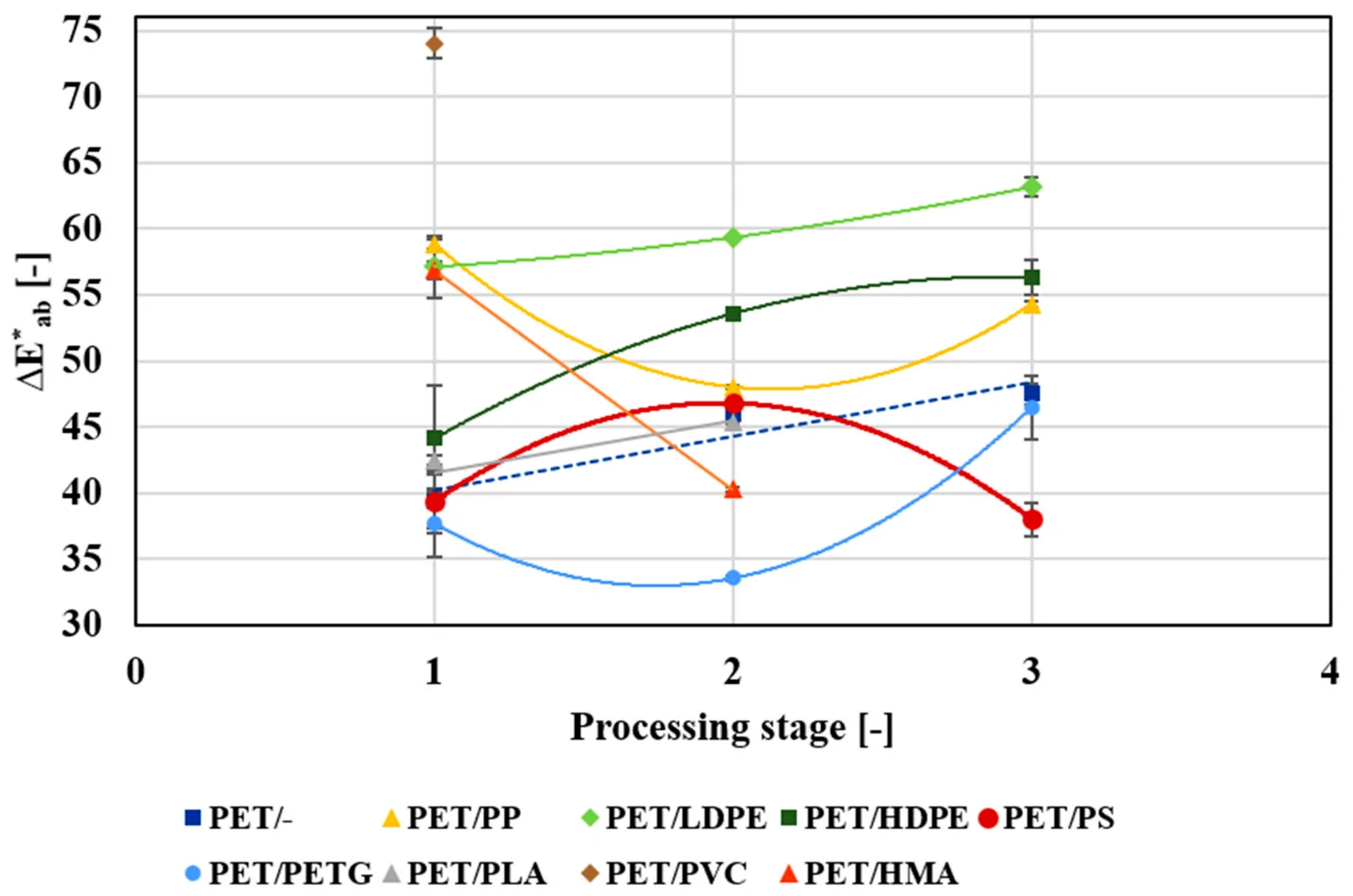
Total color difference (ΔE*ab) of the PET reference and PET/contaminant systems as a function of processing stage, calculated relative to the white reference standard (L* = 100, a* = 0, b* = 0). Values are presented as means ± standard deviation. Fifteen technical measurements were performed for each material and processing stage.

**Figure 13:**
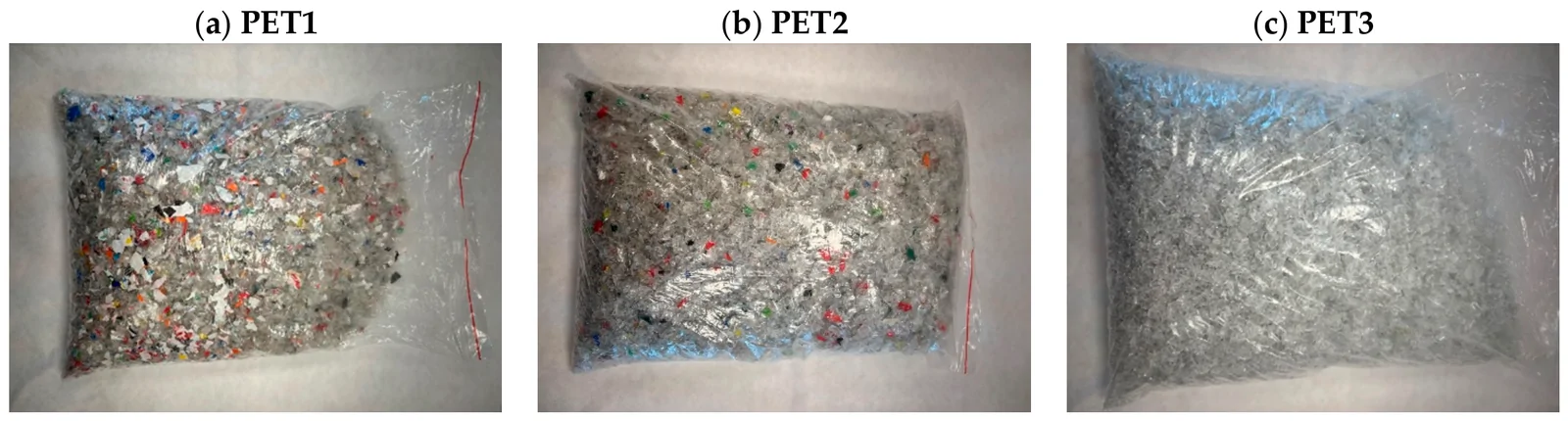
Visual appearance of industrial PET flakes collected at successive purification stages: (**a**) PET1, before ZIG-ZAG separation; (**b**) PET2, after ZIG-ZAG separation; and (**c**) PET3, after subsequent alkaline washing/decontamination.

**Figure 14:**
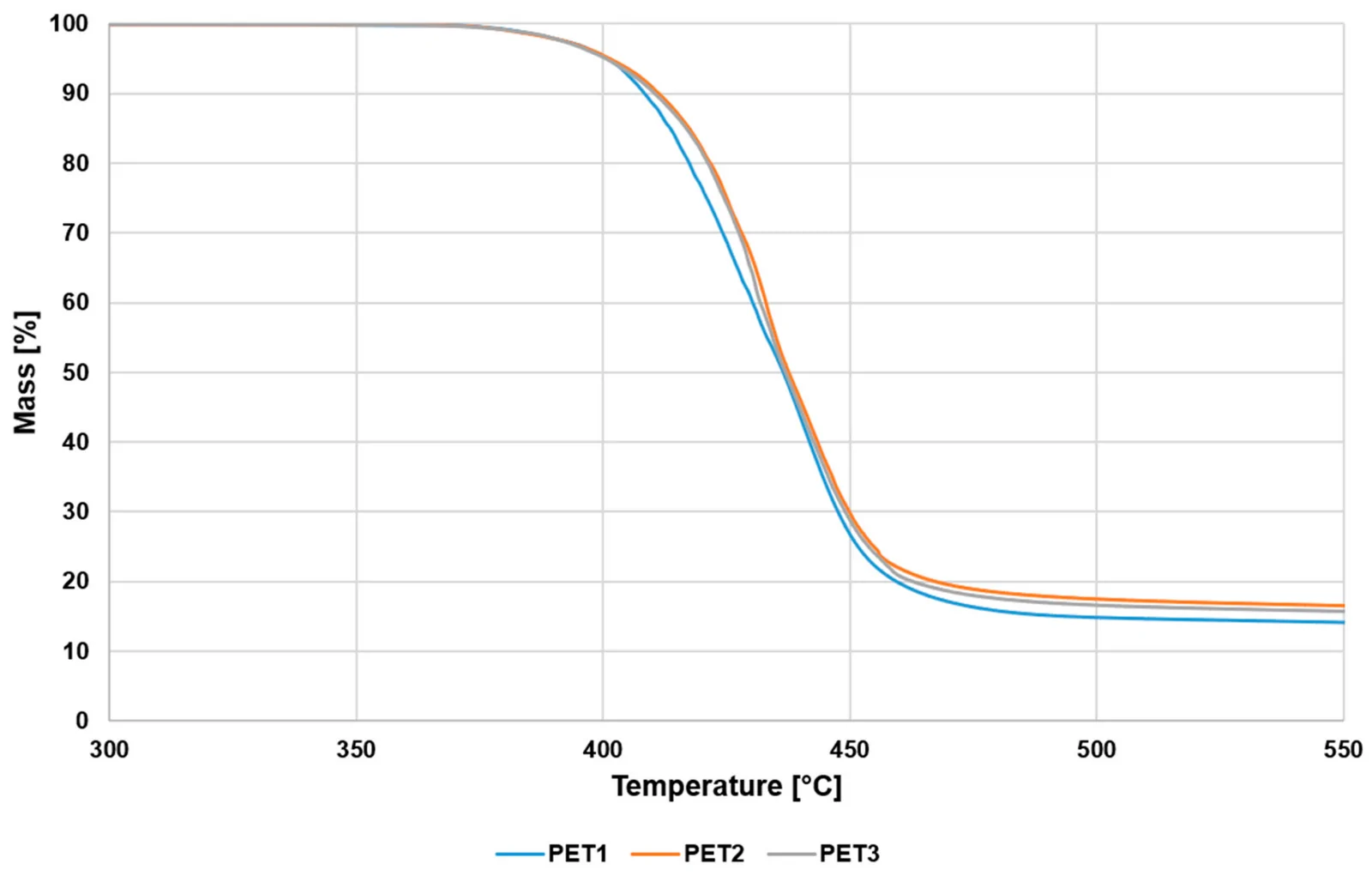
Thermogravimetric curves of industrial PET fractions collected before separation (PET1), after ZIG-ZAG separation (PET2), and after subsequent alkaline washing/decontamination (PET3).

**Figure 15:**
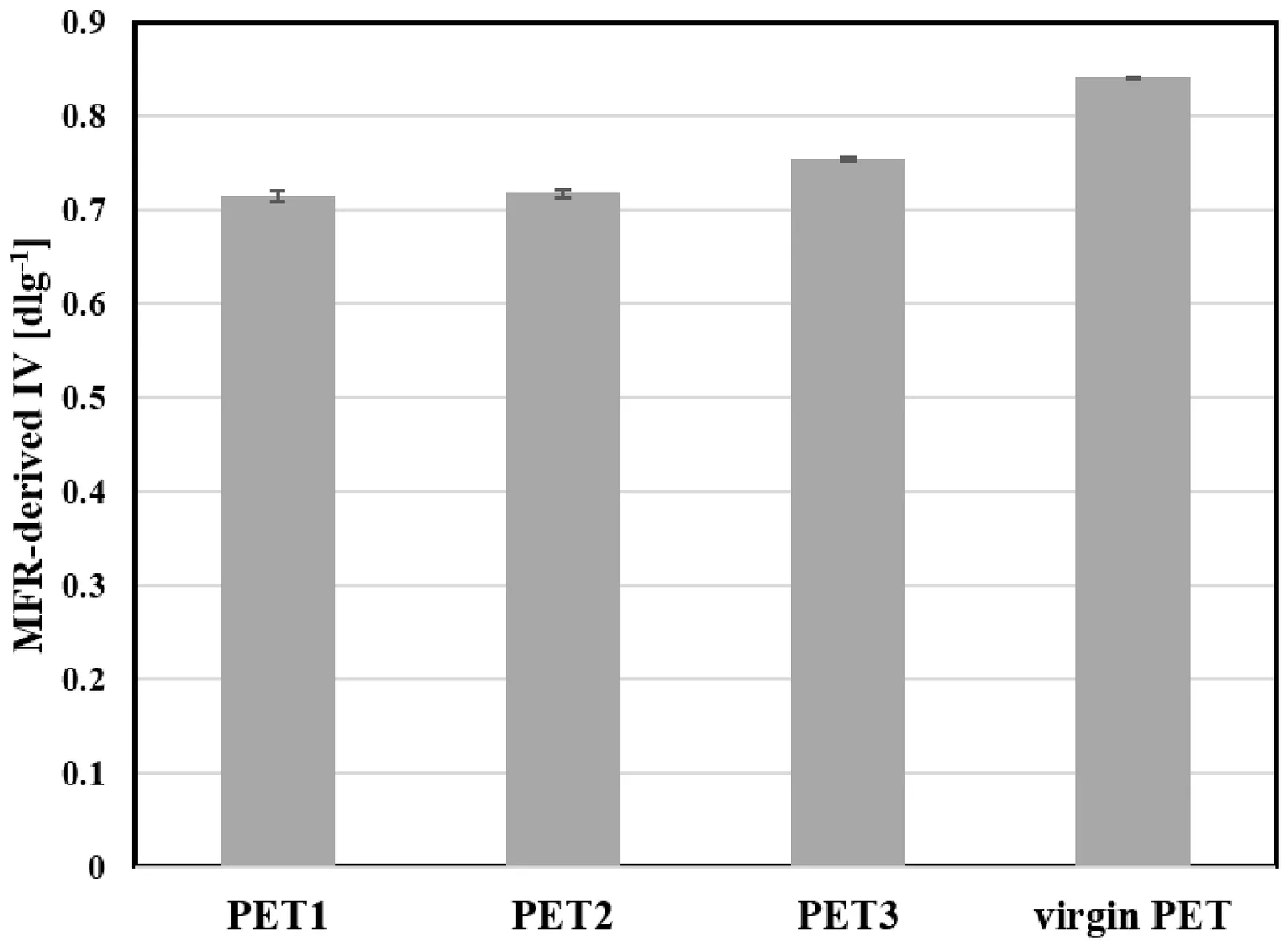
MFR-derived intrinsic viscosity (IV) of industrial PET fractions collected at successive purification stages and of the virgin PET reference.

**Table 1:** Materials used for preparation of the model PET/contaminant systems.

Material	Abbreviation	Commercial Grade/Product	Manufacturer
Polyethylene terephthalate	PET	NEOPET 80	UAB NEO GROUP, Rimkai, Lithuania
Polypropylene	PP	TATREN HM 50 46	SLOVNAFT, a.s., Bratislava, Slovakia
Low-density polyethylene	LDPE	BRALEN RB 03-23	SLOVNAFT, a.s., Bratislava, Slovakia
High-density polyethylene	HDPE	TIPELIN 1108J	MOL Petrochemicals Co. Ltd., Tiszaújváros, Hungary
Polystyrene	PS	SYNTHOS PS GP 154	SYNTHOS Kralupy a.s., Kralupy nad Vltavou, Czech Republic
Glycol-modified polyethylene terephthalate	PETG	SKYPLETE GE300	SK chemicals Co., Ltd., Seongnam, Republic of Korea
Polylactic acid	PLA	Luminy L130	TotalEnergies Corbion bv, Gorinchem, The Netherlands
Polyvinyl chloride	PVC	NERALIT 652	SPOLANA s.r.o., Neratovice, Czech Republic
Hot-melt labeling adhesive	HMA	TECHNOMELT EM 357	Henkel AG & Co. KGaA, Düsseldorf, Germany

**Note**: TECHNOMELT EM 357 was used as a representative commercial hot-melt adhesive associated with plastic bottle labeling systems.

**Table 2:** Materials identified by ATR-FTIR in the investigated commercial beverage-bottle labels.

Material Identified by ATR-FTIR	Abbreviation	Number of Labels	Fraction of Analyzed Labels (%)
Polypropylene	PP	11	78.6
Paper	—	1	7.1
Polystyrene	PS	1	7.1
Poly(ethylene terephthalate)	PET	1	7.1

## Data Availability

The original contributions presented in this study are included in the article/supplementary materials. Further inquiries can be directed to the corresponding author.

## Supplementary Materials

The following supporting information can be downloaded at: https://www.mdpi.com/article/10.3390/ma19214704/s1, Figure S1: ATR-FTIR spectra of the 14 commercial beverage-bottle labels analyzed during the initial screening; Table S1: Material identification of the commercial beverage-bottle labels analyzed by ATR-FTIR; Table S2: Melt flow rate (MFR) and MFR-derived intrinsic viscosity of the model PET/contaminant systems; Table S3: Thermal parameters of the PET/contaminant systems obtained from controlled-cooling and second-heating DSC scans; Table S4: Tensile properties of mechanically testable PET/contaminant systems; Table S5: CIE L*a*b* color parameters and total color difference in the PET/contaminant systems; Table S6: Rheological comparison of the industrial PET fractions and the virgin PET reference.
