# Supplementary material for: Effects of Packaging-Relevant Contaminants on the Property Evolution and Reprocessability of PET During Repeated Mechanical Recycling
Source: Materials (Basel). 2026 Nov 5;19(21):4704. doi: 10.3390/ma19214704 (PMC13765524; doi:10.3390/ma19214704)
Supplement: Supplementary file 1 [file materials-19-04704-s001.zip › materials-4619619-supplementary.pdf]

S1. ATR-FTIR Analysis of Commercial Beverage-Bottle Labels

ATR-FTIR spectroscopy was used to identify the material composition of 14 commercial beverage-bottle labels included in the initial screening. Measurements were performed on the unprinted side of the labels, and the obtained spectra were compared with reference spectral libraries. The material assignments are summarized in Table S1, and the corresponding ATR-FTIR spectra are presented in Figure S1.

Eleven of the fourteen analyzed labels were identified as polypropylene (PP), while one label each was identified as paper, polystyrene (PS), and poly(ethylene terephthalate) (PET). Polypropylene therefore represented 78.6% of the investigated label set. These results were used to support the selection of label-related model contaminants evaluated in the main study.

Table S1. Material identification of the commercial beverage-bottle labels analyzed by ATR-FTIR

Label designation	Identified material
**E1**	Polypropylene (PP)
**E2**	Polypropylene (PP)
**E3**	Polypropylene (PP)
**E4**	Polypropylene (PP)
**E5**	Polypropylene (PP)
**E6**	Polypropylene (PP)
**E7**	Polypropylene (PP)
**E8**	Polypropylene (PP)
**E9**	Polypropylene (PP)
**E10**	Polypropylene (PP)
**E11**	Polypropylene (PP)
**E12**	Paper
**E13**	Polystyrene (PS)
**E14**	Poly(ethylene terephthalate) (PET)

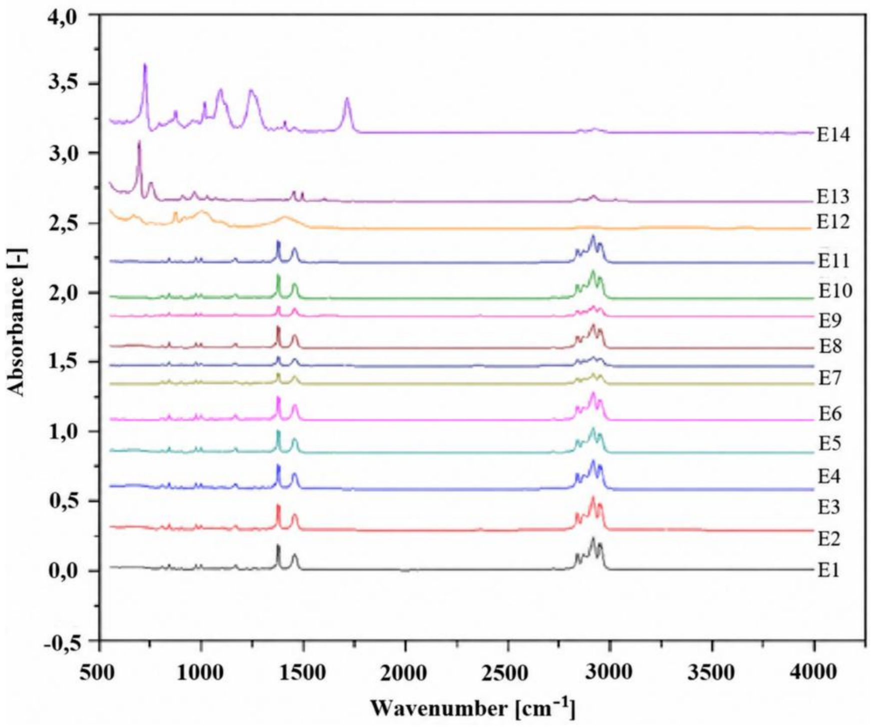

Figure S1. ATR-FTIR spectra of the 14 commercial beverage-bottle labels analyzed during the initial screening.

S2. Additional Numerical Data

The following tables provide numerical values corresponding to the datasets presented graphically in the main manuscript and facilitate direct comparison among the investigated PET/contaminant systems.

Table S2. Melt flow rate (MFR) and MFR-derived intrinsic viscosity of the model PET/contaminant systems.

System	Processing stage	MFR [g/10 min]	MFR-derived IV [dL g^{-1}]
PET	P1	20.70	0.837
PET	P2	40.80	0.721
PET	P3	77.20	0.638
PET/PP	P1	57.56	0.665
PET/PP	P2	103.80	0.574
PET/PP	P3	149.67	0.526
PET/LDPE	P1	66.74	0.640
PET/LDPE	P2	89.70	0.596
PET/LDPE	P3	115.10	0.560
PET/HDPE	P1	54.04	0.674
PET/HDPE	P2	86.60	0.598
PET/HDPE	P3	118.17	0.556
PET/PS	P1	45.65	0.703
PET/PS	P2	91.79	0.592
PET/PS	P3	139.00	0.534
PET/PETG	P1	71.84	0.629
PET/PETG	P2	120.67	0.553
PET/PETG	P3	170.87	0.508
PET/PLA	P1	89.06	0.596

PET/PLA	P2	164.13	0.513
PET/PVC	P1	2201.37	0.303
PET/HMA	P1	161.43	0.515
PET/HMA	P2	258.23	0.459

Values correspond to the mean values used in the revised manuscript. The PET reference values are the manuscript dataset (MFR: 20.7, 40.8, and 77.2 g/10 min; MFR-derived IV: 0.837, 0.721, and 0.638 dL g^{-1} for P1–P3). Point-specific uncertainty is shown in the Figures 1 and 2.

Table S3. Thermal parameters of the PET/contaminant systems obtained from controlled-cooling and second-heating DSC scans.

System	Stage	Tg [°C] 2nd heat	Tc [°C] cooling	Tm [°C] 2nd heat	ΔHm [J/g] 2nd heat	χc [%]
PET	P1	81	183	251	33.48	23.91
PET	P2	81	199	253	32.87	23.48
PET	P3	79	204	252	37.66	26.90
PET/PP	P1	79	195	250	34.40	25.33
PET/PP	P2	79	200	251	34.99	25.77
PET/PP	P3	80	203	252	37.68	27.75
PET/LDPE	P1	82	187	254	31.03	22.85
PET/LDPE	P2	82	198	255	32.37	23.84
PET/LDPE	P3	80	203	253	36.27	26.71
PET/HDPE	P1	70	188	253	34.01	25.04
PET/HDPE	P2	81	199	252	35.61	26.22
PET/HDPE	P3	80	202	251	34.32	25.27
PET/PS	P1	86	187	251	34.94	25.73
PET/PS	P2	79	200	252	34.92	25.71
PET/PS	P3	77	204	251	33.62	24.76
PET/PETG	P1	79	192	250	28.59	21.05
PET/PETG	P2	79	201	251	30.83	22.70
PET/PETG	P3	78	204	250	32.53	23.95
PET/PLA	P1	77	195	251	35.57	26.19
PET/PLA	P2	-	203	250	34.26	25.23
PET/PVC	P1	-	206	248	54.36	40.03
PET/HMA	P1	85	199	252	36.15	26.62
PET/HMA	P2	82	203	253	31.74	23.37

The degree of crystallinity was calculated for the PET phase using ΔHm° = 140 J g^{-1} and correcting for the PET mass fraction (1.00 for PET and 0.97 for contaminated blends).

Table S4. Tensile properties of mechanically testable PET/contaminant systems.

System	Stage	Tensile strength at break, σB [MPa]	Elongation at break, εB [%]
PET	P1	48.87	163.16
PET	P2	45.64	119.01
PET	P3	45.26	118.15
PET/PP	P1	40.35	20.16
PET/PP	P2	47.72	13.47
PET/PP	P3	32.20	6.66
PET/LDPE	P1	40.03	33.12
PET/LDPE	P2	52.88	14.65
PET/LDPE	P3	46.80	17.08
PET/HDPE	P1	46.95	16.97

PET/HDPE	P2	44.68	23.01
PET/HDPE	P3	65.49	9.81
PET/PS	P1	42.61	28.29
PET/PS	P2	41.21	33.42
PET/PS	P3	39.51	125.40
PET/PETG	P1	47.23	23.88
PET/PETG	P2	54.66	16.55
PET/PETG	P3	36.96	163.78

Five strand specimens were tested for each material and processing stage. PET/PLA, PET/PVC, and PET/HMA are not included because the extruded strands fractured during specimen mounting and valid tensile measurements could not be obtained.

Table S5. CIE L*a*b* color parameters and total color difference of the PET/contaminant systems.

System	Stage	L*	a*	b*	ΔE*ab
PET	P1	60.86 ± 7.89	-1.23 ± 1.01	-3.66 ± 1.73	39.39 ± 7.83
PET	P2	54.10 ± 2.60	-2.29 ± 0.14	-1.06 ± 0.88	45.98 ± 2.61
PET	P3	53.62 ± 2.83	-0.32 ± 0.25	10.14 ± 0.39	47.48 ± 2.79
PET/PP	P1	41.69 ± 1.29	-0.18 ± 0.19	-8.02 ± 0.69	58.86 ± 1.26
PET/PP	P2	52.53 ± 6.59	-1.79 ± 0.49	-6.99 ± 1.59	48.03 ± 6.72
PET/PP	P3	45.82 ± 1.01	-1.31 ± 0.32	-3.21 ± 0.52	54.29 ± 1.02
PET/LDPE	P1	43.16 ± 9.09	-0.25 ± 1.33	-4.19 ± 3.38	57.12 ± 8.94
PET/LDPE	P2	40.84 ± 2.55	-0.08 ± 1.05	-4.21 ± 2.06	59.36 ± 2.41
PET/LDPE	P3	36.88 ± 2.63	0.35 ± 0.34	2.32 ± 1.24	63.18 ± 2.59
PET/HDPE	P1	56.85 ± 15.66	-1.58 ± 1.56	-8.48 ± 2.14	44.12 ± 15.19
PET/HDPE	P2	46.83 ± 3.19	-0.04 ± 1.77	-0.16 ± 6.68	53.58 ± 2.98
PET/HDPE	P3	44.00 ± 5.18	0.84 ± 0.82	3.40 ± 4.96	56.35 ± 4.81
PET/PS	P1	61.74 ± 9.10	-2.92 ± 0.70	-8.63 ± 2.07	39.61 ± 6.94
PET/PS	P2	54.13 ± 7.32	-3.51 ± 0.73	-3.67 ± 3.61	46.80 ± 7.24
PET/PS	P3	62.15 ± 4.91	-2.30 ± 0.33	-2.83 ± 2.56	38.22 ± 4.79
PET/PETG	P1	62.21 ± 10.12	-1.88 ± 1.39	-6.90 ± 4.12	37.72 ± 10.12
PET/PETG	P2	67.06 ± 12.61	-0.88 ± 1.89	0.28 ± 4.93	33.55 ± 11.99
PET/PETG	P3	53.77 ± 9.44	-2.26 ± 1.27	-2.55 ± 3.26	46.47 ± 9.48
PET/PLA	P1	58.18 ± 1.43	-1.16 ± 0.14	-7.64 ± 0.26	42.54 ± 1.40
PET/PLA	P2	55.30 ± 1.46	-1.28 ± 0.13	-7.17 ± 0.24	45.30 ± 1.44
PET/PVC	P1	27.62 ± 4.13	1.54 ± 1.01	15.49 ± 3.05	74.36 ± 4.33
PET/HMA	P1	43.18 ± 2.54	0.06 ± 0.76	1.60 ± 2.51	56.90 ± 2.45
PET/HMA	P2	59.82 ± 4.24	-2.83 ± 0.22	-0.52 ± 0.57	40.29 ± 4.24

Values are mean ± standard deviation. Color characterization consisted of 15 technical measurements per material and processing stage.

Table S6. Rheological comparison of the industrial PET fractions and the virgin PET reference.

Fraction	Process-stage description	MFR [g/10 min]	MFR-derived IV [dL g^{-1}]
PET1	Before ZIG-ZAG separation	42.73	0.714
PET2	After ZIG-ZAG separation	41.35	0.717
PET3	After separation and alkaline washing/decontamination	34.27	0.754
virgin PET	Virgin PET reference	22.03	0.841

Values correspond to the curated averages used in Figure 15 of the main manuscript.
